# Schistosomiasis Drug Discovery in the Era of Automation and Artificial Intelligence

**DOI:** 10.3389/fimmu.2021.642383

**Published:** 2021-05-31

**Authors:** José T. Moreira-Filho, Arthur C. Silva, Rafael F. Dantas, Barbara F. Gomes, Lauro R. Souza Neto, Jose Brandao-Neto, Raymond J. Owens, Nicholas Furnham, Bruno J. Neves, Floriano P. Silva-Junior, Carolina H. Andrade

**Affiliations:** ^1^ LabMol – Laboratory for Molecular Modeling and Drug Design, Faculdade de Farmácia, Universidade Federal de Goiás – UFG, Goiânia, Brazil; ^2^ LaBECFar – Laboratório de Bioquímica Experimental e Computacional de Fármacos, Instituto Oswaldo Cruz, Fundação Oswaldo Cruz, Rio de Janeiro, Brazil; ^3^ Diamond Light Source Ltd., Didcot, United Kingdom; ^4^ Research Complex at Harwell, Didcot, United Kingdom; ^5^ The Rosalind Franklin Institute, Harwell, United Kingdom; ^6^ Division of Structural Biology, The Wellcome Centre for Human Genetic, University of Oxford, Oxford, United Kingdom; ^7^ Department of Infection Biology, Faculty of Infectious and Tropical Diseases, London School of Hygiene and Tropical Medicine, London, United Kingdom

**Keywords:** schistosomiasis, drug discovery, artificial intelligence, fragment-based drug discovery, phenotypic screening, target-based screening

## Abstract

Schistosomiasis is a parasitic disease caused by trematode worms of the genus *Schistosoma* and affects over 200 million people worldwide. The control and treatment of this neglected tropical disease is based on a single drug, praziquantel, which raises concerns about the development of drug resistance. This, and the lack of efficacy of praziquantel against juvenile worms, highlights the urgency for new antischistosomal therapies. In this review we focus on innovative approaches to the identification of antischistosomal drug candidates, including the use of automated assays, fragment-based screening, computer-aided and artificial intelligence-based computational methods. We highlight the current developments that may contribute to optimizing research outputs and lead to more effective drugs for this highly prevalent disease, in a more cost-effective drug discovery endeavor.

## Introduction

Schistosomiasis is a neglected tropical disease (NTD) caused by trematode parasites belonging to the genus *Schistosoma*. The most clinically-relevant species are *S. mansoni*, *S. japonicum* and *S. haematobium* while *S. mekongi, S. guineensis* and *S. intercalatum* have lower prevalence (1, 2). According to the World Health Organization (WHO), approximately 229 million people are infected worldwide, causing around 200,000 deaths annually (2). However, this is probably an underestimation, due to the low sensitivity of the available diagnostic methods to detect low intensity parasite infections (3, 4). It ranks second behind malaria in terms of prevalence and socioeconomic impact, causing the loss of more than 2.6 million disability-adjusted life years (DALYs) (5).

Humans become infected when the cercariae larvae, released by the snail intermediate hosts, penetrate through the skin during contact with contaminated freshwater (1, 6). Then, cercariae access the host circulation and develop into juvenile and adult worms (7). Paired female and male adult schistosomes live in the blood vessels where they produce eggs that are excreted in faeces (*S. mansoni, S. japonicum, S. intercalatum, S. guineenses* and *S. mekongi*) or urine (infections by *S. haematobium*) (6). Eggs become trapped in human tissues causing inflammatory immune reactions (including granulomata) that damage organs resulting in intestinal, hepatosplenic, or urogenital disease (8). The eggs that reach the environment, hatch in the water and release the larval stage miracidia. The Miracidia infect the intermediate hosts and continue the parasite’s life-cycle (9).

Because the development of a schistosomiasis vaccine has proved challenging (10, 11), the treatment and control of schistosomiasis continue to depend, on the almost 50-year-old drug, praziquantel (PZQ) (12, 13). PZQ is generally effective against adult and schistosomula stages of all schistosome species and is well tolerated, causing only mild and transient side effects (14, 15). However, PZQ is ineffective against juvenile schistosomes, which contributes to the failure of the drug to cure the disease and the need for new rounds of treatment (16, 17). Moreover, PZQ is administered as a racemic mixture, wherefore only half of PZQ dose (i.e., *R*-PZQ stereoisomer) is pharmacologically active. The *S*-PZQ, besides being pharmacologically inactive, contributes to the bitter taste and the large size of PQZ tablets, both of which decrease patient compliance and are not suitable for children (18, 19). Moreover, PZQ has been used in mass drug administration campaigns for many decades and this may account for a selection pressure that can promote the development of parasitic resistance (20). In fact, reduced PZQ efficacy has been demonstrated both in laboratory and field isolates (21–28). Consequently, there is an urgent need for new antischistosomal drugs.

One of the foremost challenges to the discovery of new anti-schistosomal drugs is the long and complex life cycle of the parasite, which makes screening campaigns technically difficult (29). The phenotypic screening of whole-organisms *in vitro* and/or in animal models is the approach that is most used for finding hit compounds (i.e., active compounds *in vitro* based on a defined activity threshold) (30–32), though animal models tend to be costly and time-consuming (33). These assays require the maintenance of the parasites’ life cycle – including both the intermediate (snails) and the definitive hosts (hamsters or mice) – in order to have regular access to parasites. However, these laboratory-based life cycles can only produce a restricted number of adult-stage schistosomes (34). As a consequence, most early compound screening efforts use newly transformed schistosomula (NTS), which are obtained from the mechanically transformed cercariae (35). This can limit the finding of new hits, as the sensitivity to compounds can vary between life cycle stage and gender of the parasite (36, 37).

Screening compounds for anti-schistosomal activity is typically carried out manually where an analyst identifies the 5presence of morphological or behavioral changes in the parasites by microscopy (29). A numerical scale (“severity score”), usually including four (38, 39) or five (29, 40) scores, is used to describe the phenotypes. However, this analysis is subjective, semi-quantitative, time-consuming and the results can vary largely from analyst to analyst (41–43). Nonetheless, severity scoring systems have been successful in identifying hits, defining SAR, and identifying compounds that translate with *in vivo* efficacy in the mouse model of *S. mansoni* infection (44).

In this review we focus on innovative approaches to the identification of antischistosomal drug candidates, including the use of automated assays, fragment-based screening, computer-aided and artificial intelligence-based computational methods. We also highlight the current developments that may contribute to optimizing research outputs and lead to more cost-effective drugs for this highly prevalent disease.

## Phenotypic Screening

Phenotypic screening consists of testing substances that could possibly cause phenotypic changes considered relevant to a biological system. Phenotypic-based drug discovery (PDD), compared to target-based drug discovery (TDD), has the advantage of presenting a greater probability of identifying compounds which will be translated to *in vivo* tests since they better mimic the complex environment of living systems. For example, in a cellular assay, a test compound may have to cross cellular membranes and/or resist degradation by metabolic enzymes before interacting with its target(s). These factors may have a significant impact in a compound’s biological activity and are not taken into account in a target-based assay. Hence, a hit coming from a phenotypic screen has much more biological value than one coming from a target-based screen (45, 46). Nonetheless, an important consideration must be given to the higher cases of false negatives in PDD campaigns when compared to TDD. As discussed by Geary and colleagues (33), the false negatives result from the inability of some compounds to reach a proper concentration inside a whole organism, which may hinder the detection of potential schistosomicidal molecules.

PDD approach tends to be more time-consuming and costly to develop and run than TDD (47). This is mainly due to the implementation of higher complexity screening assays, and to other factors such as the parallel use of genetic and small molecules screens, as well as more complicated hit validation and target identification efforts (48). Furthermore, the establishment of structure-activity relationship (SAR) is more challenging due to other concurrent factors, such as membrane permeability and off-target binding, though there are several examples of successful SAR studies using schistosome phenotypic assays in the literature (49–51). Hence, PDD approach is limited in this sense due to unknown mechanism of action, potentially targeting different types of proteins, such as receptors, enzymes, transcription factors and even different signaling pathways (45). Additional assays may be necessary to support the SAR in PDD approaches. On the other hand, the multiplicity of potential targets in PDD can be a source of serendipity (33, 46). In contrast, the prior knowledge of the target in TDD helps to accelerate the interpretation of SAR data. In spite of their differences, it is increasingly known that PDD and TDD must be seen as complementary in the R&D of new drugs (48, 52, 53).

In the following topics we will address some of the main phenotypic assays that have been used for schistosomiasis drug discovery. They consist in labelled (employing fluorescent or luminescent dyes) and label-free assays that are able to detect drug-induced phenotypes in different stages of the parasite, such as schistosomula (NTS), juvenile (1-5 weeks post infection) and adult (6-7 weeks or over post infection) (38).

### Luminescence- and Fluorescence-Based Phenotypic Assays

Some methodologies used in schistosome phenotypic screening are based on fluorescent or luminescent dyes commonly employed in cellular viability/cytotoxicity assays (**Table 1**). Propidium iodide (PI), a DNA intercalator, for instance, is a fluorescent dye that is not able to cross membrane cells and can only stain nucleic acids of cells that have lost their membrane integrity. For this reason, such fluorophore is used to differentiate living and dead cells. Unlike PI, fluorescein diacetate (FDA) can cross biological membranes, and is converted by healthy cells into fluorescein, another fluorescent dye. Peak et al. (54) developed a microplate-based assay to measure schistosomula viability using PI/FDA. In this method, the fluorescence emitted by FDA and PI stained parasites is quantified by a microplate reader and later converted into worm viability using the fluorescence ratio FDA/(PI + FDA). This assay was able to detect the effect of some known schistosomicidal drugs, namely auranofin, gambogic acid and amphotericin b, but failed in measuring the effect of praziquantel and other compounds previously identified as actives by microscopy (55). Braun et al. (56) also used PI and FDA probes in the development of a quantitative HTS (qHTS) fluorescent-based bioassay to identify schistosomula in water samples (56). The results obtained with this method showed no statistically significant differences when compared to visual inspections carried out by manual microscopy.

**Table 1:** Summary of some luminescence and fluorescence schistosomal drug assay methodologies.

Assay type	Marker type	Principle of methods	Method	Suitable for screening	Reference
**PI^a^/FDA^b^**	Viability and cytotoxicity	PI: stain nucleic acids in damaged worms FDA: cleaved by esterases in healthy worms	Fluorescence-based	Yes	(54)
**CellTiter-Glo^®^**	Viability	Luciferin is oxidized by luciferase in the presence of worms’ ATP	Luminescence-based	Yes	(38)
**Resazurin**	Viability	Metabolic reduction of resazurin	Fluorescence-based	No	(39, 42)

^a^Propidium iodide; ^b^Fluorescein diacetate.

Mansour et al. (42) used a commercial solution of resazurin (Alamar Blue^®^), a redox-sensitive probe, to measure the viability of schistosomula in microplates. This assay is based on the principle that only metabolic active worms can reduce resazurin to resorufin, a fluorescent molecule. During validation, this assay proved to be useful in evaluating the effect of compounds that kill or provoke severe damage to the parasite (e.g., oltipraz), but showed less sensitivity towards those that elicited more subtle effects (e.g., praziquantel) detectable by conventional microscopy.

Lalli et al. (38) developed and validated a luminescence-based method for the evaluation of schistosomula viability by quantifying ATP (38). This assay is carried out using a commercial kit (CellTiter-Glo^®^) which contains luciferase and its substrate luciferin as the main components. In principle, metabolic active worms produce ATP which participates in the reaction catalysed by luciferase. As a result, a luminescence signal is produced and registered in a microplate reader. This medium-throughput method is suitable for semi-automated screening of chemical libraries and has several benefits such as speed in screening, reproducibility and non-subjectivity. Guidi et al. (57) used the same luminescence-based technique, combined with HTS, to search for molecules with schistosomicidal activity. As a result, a few compounds capable of impairing the viability of the larval, juvenile, and adult phases were identified, with potency against juveniles higher than PZQ. In addition, changes in egg formation and production are among the phenotypic modifications.

However, despite its success in generating dose-response curves for some known schistomicidal drugs (e.g., gambogic acid and oltipraz) this method was unable to detect the effect of praziquantel and oxamniquine on schistosomula viability. Maccesi et al. (44) also could not detect the biological effect of some schistomicidal compounds using another phenotypic assay. In their work, *S. mansoni* schistosomula were screened with the 400 compounds of the MMV Pathogen Box in three different institutions. Two of them employed visual scoring (microscopy) to describe drug-induced phenotypes while the other used a colorimetric assay based on the metabolic reduction of XTT to measure parasites viability. In nearly 74% of the cases, all three methods agreed on the classification of the compounds (active/inactive) after 72h of incubation. Nonetheless, unlike the visual methods, the XTT-based assay did not identify PZQ and other compounds (e.g., auranofin, nitazoxanide) as actives against schistosomula. This may be due to the fact that metabolic-based assays were originally designed to be used in cell and unicellular organisms. Its use in multicellular and more complex models are prone to missing important phenotypes (44) found in these organisms, like the dysregulation of the neuromuscular system, a mechanism attributed to PZQ (58).

Panic et al. (39) performed a review of fluorescent and luminescent markers used on *S. mansoni* drug assays and confirmed Lalli et al. (38) studies of development and validation of a luminescence-based assay (CellTiter-Glo^®^). In contrast to resazurin assays, which are also used to determine the viability of *Schistosoma* parasites, CellTiter-Glo^®^ was able to determine the IC_50_ of mefloquine and to detect the schistomicidal activity of some FDA-approved compounds, showing results that correlate with microscopic findings.

The assays described in this topic represent a major advance in schistosomiasis drug discovery. Nonetheless, they show some limitations that must be taken into consideration. Compared to colorimetric methods, such as XTT-based assay, fluorescence/luminescence-based assays require black/white microplates which are more expensive (59). In some cases, a given method may not detect the schistomicidal effect of a compound recognized as active by other techniques, including conventional microscopy (39). Moreover, some test compounds may interfere with the assay (e.g., compounds auto-fluorescence), leading to a misinterpretation of the results (38). Therefore, it is advisable to be cautious when considering the results obtained from a single method, being recommended to corroborate the results using at least one orthogonal assay.

### Label-Free Automated Phenotypic Assays for Schistosomiasis Drug Discovery

**Figure 1:**
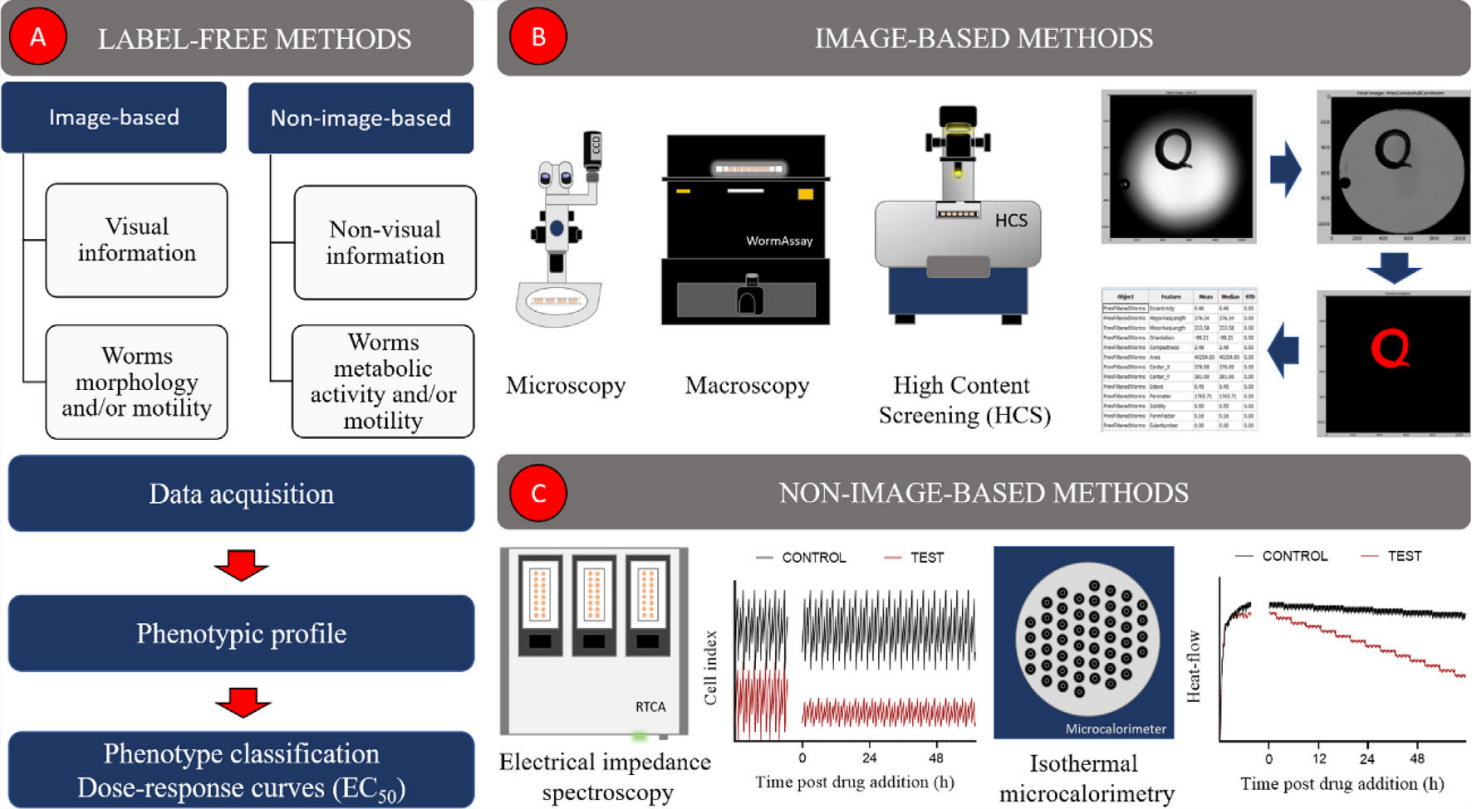
Label-free image-based and non-image-based automated methods used in schistosomiasis drug discovery. **(A)** Overall description of label-free methods, **(B)** image-based methods, **(C)** non-image-based methods.

Label-free automated assays detect phenotypic alterations in schistosomes in the absence of any kind of label (e.g., fluorescent probe). In general, they can be divided into two main groups: image-based (most common) (60–63) and non-image-based methods (64, 65) (**Figure 1A**). The former use visual information, such as morphology and/or motility of the parasite, to describe a phenotype (**Figures 1A, B**). In contrast, non-image-based methods, herein exemplified by electrical impedance spectroscopy (65) and microcalorimetry (64), create phenotypic profiles based on worm’s metabolic activity and/or its motility (**Figures 1A, C**). Image-based and non-image-based assays have been largely employed in drug discovery campaigns to identify new schistosomicidal compounds and calculate their potencies. Some of these methods and their main characteristics (e.g., type of readout, assay format and the number of parasites required per assay) are shown in **Table 2** and will be discussed in more detail in the following section.

**Table 2:** Label-free automated assays used in schistosomiasis drug discovery.

Assay	Assay measurements	Readout	Development stage of schistosome	Assay format	Main hardware	Number of parasites per assay
*Image-based assays*						
Video microscopy (62, 66, 67)	Parasites motility (62)	Light microscopy images	Schistosomula	24-well microplate	Microscope equipped with a digital camera	30-40
	Parasites motility (66)		Adult	12-well microplate		5 pairs
	Parasites motility (67)			6-well microplate		4-5 pairs
QDREC (68)	Parasite’s morphology		Schistosomula	96-wells microplate		400
WormAssay (60)	Parasite’s motility	Light macroscopy images	Adult	6-96-wells microplate	Custom-made camcorder-based system	1 or more
High Content Screening (HCS) (61, 69, 70)	Parasites motility/morphology (61, 70)	Light microscopy images	Schistosomula	384-wells microplate (70)	HCS system	120
			Schistosomula	U-bottom 96-wells microplate (61)		40
	Parasites motility (69)		Adult	96-wells microplate		1
*Non-image-based assays*						
xWORM (71)	Parasite’s motility	Electrical impedance	Adult	E-plates	Real time cell analysis (RTCA) system	1
			Cercariae			562
	Egg hatching		Eggs			5000
Electrical-impedance microwell (EIM) platform (65)	Parasite’s motility	Electrical impedance	Schistosomula	Custom-made plate containing 32 analysis units	Custom-made EIM parallelized platform	10-15
Isothermal microcalorimetry (64)	Parasites metabolic activity/motility	Heat-flow	Schistosomula	1 mL glass ampoules	Isothermal microcalorimeter	400-1000
			Adult			3-4

#### Image-Based Methods

The automatic quantification of phenotypic features from schistosomes images has been addressed in several ways (60–63, 66, 69, 70, 72). Most assays rely on home-made (60) or commercial (63, 70) systems, equipped with digital cameras/camcorders to acquire images of unstained parasites in microplates. Image analysis is carried out, using either commercial (62, 69, 70) or custom-built (60, 68) software and generally consists of three main steps: image processing, parasite(s) detection and features extraction (**Figure 1B**). By the end of the analysis, a phenotypic profile, composed of a variable number of features, is created for one (63, 69, 70) or more (60) worms. These profiles can be used to identify schistosomicidal compounds, measure their effect and potency, as well compare their responses to those elicited by other compounds (60, 63, 69, 70).

One common strategy to acquire schistosome images is by video microscopy. Ribeiro and colleagues implemented two assays using this technique to measure the motility of NTS (62) and adult (66) forms of *S. mansoni*. In these assays, parasites are distributed in microplates and their videos recorded over a few minutes. Image frames are analyzed in ImageJ (73), an open-source software. For NTS analysis, worms are detected as ellipsoid objects and their body length are estimated by the size of the major axis of the ellipses. NTS movements (shortening and elongation of the body) are calculated by the frequency of length changes over time (62). For adult analysis, each image frame is submitted to a series of pre-processing steps (e.g., illumination correction and background removal) before representing the worms as binary objects. Then, the difference in pixels between pairs of consecutive images are measured throughout the entire video. The subtracted pixels represent a change in parasite position over time and are used as a metric to measure worm´ motility (66). These methods were employed to evaluate the effect of different compounds on schistosome NTS/adults worms, such as tyrosine-derived signaling agonists/antagonists (74), natural alkaloids (75–77) and analogs (77, 78), as well as others with biological activity in humans (e.g., NPS-2143, a calcium-sensing receptor antagonist) (77).

McCusker and colleagues (67, 72) also applied video microscopy to record morphological and motility aspects of adult schistosomes. In their assay, 1 min videos of adult worms, distributed in 6-well dishes, are acquired, and saved as images Z-stacks. During image analysis in ImageJ, maximum intensity projections are generated to each Z-stack resulting in a composite image for which pixel integrated pixel values are measured. This metric represents the total movement of the parasite over time and can be used to compare treated and non-treated parasites. This method detected the schistosomicidal effect of non-sedating benzodiazepines (72) and FPL-64176 (67), a human L-type Ca^2+^ channel agonist.

WormAssay is a home-made low-cost solution to screen compounds against adult schistosomes (and other macroscopic parasites) developed by Marcellino et al. (60). This system consists of two devices: an imaging apparatus and a Mac computer (image acquisition control and analysis). The former can be described as a light-tight box containing a high-definition video (HDV) camcorder mounted at the bottom and a hinge lid at the top harboring the microplate chamber. Inside the chamber a white LED strip is used to laterally illuminate the microplate. During acquisition, dark-field videos of 0.5 – 1 min length are recorded from the entire microplate. Image analysis is performed in real-time using a custom open-source free software (Mac application) installed in the computer. The overall motility of worms, measured for each well, is determined by two algorithms: one that calculates the average velocity of moving contours inside the well and another which detects changes in the occupation and vacancy of pixels between a group of frames. WormAssay quantified the effect of several compounds on worm motility, including neuromodulatory drugs (79), phenylpyrimidines (80) and inhibitors of *S. mansoni* cyclic nucleotide phosphodiesterase 4 (*Sm*PDE4) (81) and proteasome (82). Recently, a modified version of WormAssay software, named WormAssayGP2, was released by Padalino and colleagues (83, 84) and contains minor modifications related to the source code and user interface (85). To date, WormAssayGP2 has been used to detect the schistomicidal activity of putative inhibitors of *S. mansoni* lysine specific demethylase 1 (*Sm*LSD1) (86) and histone methyltransferase mixed lineage leukemia-1 (*Sm*MLL-1) (84), as well as human ubiquitin-proteasome system (85) inhibitors.

One of the first attempts to describe complex phenotypes is schistosomes was carried out by Singh et al. (87). They developed an automated method to detect, track and classify individual NTS in microscopy videos. Since then, several modifications have been made regarding worms segmentation (88) and phenotypic analysis (63, 68, 89, 90). The algorithms developed in these studies have proved to be useful in quantifying the schistosomicidal activity of PZQ (63), chlorpromazine (63), statins (91) and inhibitors of human polo-like kinase 1 (PLK1) (89, 92). They have also been implemented in the “quantal dose-response calculator” (QDREC), a web server that automatically extracts quantal time- and dose-response information from bright-field images of schistosomes NTS (or other parasites) (68). QDREC quantifies 71 image-based features related to the appearance, shape and texture, of each segmented parasite. These features serve as inputs for supervised machine learning algorithms which classify parasites as “normal” or “degenerate”. The proportion of “degenerate” worms in a well is employed as a metric to estimate the schistosomicidal effect of a given compound and can be used to create dose-response curves. QDREC was validated with 12 schistosomicidal compounds (e.g., statins, PZQ, closantel, niclosamide and sorafenib) showing comparable results with visual annotation for both parasite classification and dose-response curves.

Paveley et al. (70) was a pioneer in implementing automated microscopy, also known as high content screening (HCS), to extract multivariate data from schistosomes. They created an HCS-based automated platform to identify active compounds on NTS of *S. mansoni*. In this assay, a HCS system collects bright-field images of each well of a 384-wells microplate through two distinct modes: a time-lapse image acquisition (5x6 s interval) using a 4x objective, for motility analysis, and one acquisition of four adjacent images using a 10x objective, for morphology measurements. Image analysis is performed in Pipeline Pilot 8.5 software (Accelrys Inc., San Diego, USA) and includes a series of sequential image operations, such as thresholding, filtering, detecting boundaries prior worm´s segmentation. Morphological (e.g., area, texture, pixel intensity) and motility-related features are quantified for each NTS and summarized into a phenotype (processed through Bayesian models) and a motility score, respectively. The final scores are obtained by averaging the scores of all parasites inside each well. Test compounds are declared “hits” (i.e., actives) if phenotype and motility scores exceed a defined threshold for each metric. This assay was able to detect the anti-worm effect of known schistosomicidal drugs (oltipraz and dihydroartemisinin) (70), several drug candidates from small (93, 94) and large (70, 95) chemical libraries, as well FDA-approved drugs (e.g., kinase inhibitors) (96). This analysis has also been employed by Hoffmann and colleagues (84, 86, 97–100) who measured the schistosomicidal activity of plant-derived compounds [e.g., diterpenoids (97, 99) and triterpenoids (100)] and of potential inhibitors of *S. mansoni* histone-modifying enzymes (40, 84, 86, 98).

Recently, Chen et al. (61) developed another HCS-based high-throughput assay to extract phenotypic features from treated and non-treated NTS. It consists of a fully integrated platform that can perform multiple automated operations ranging from liquid handling of NTS suspensions to image acquisition/analysis. The latter tasks are carried out as follows: time-lapse bright-field images (30 x 0.66 s interval) are acquired from each well of a U-bottom 96-wells microplate using a 10x objective in a HCS system. During image analysis, each NTS is segmented and classified as “clear” (normal) or “degenerate” (damaged/dying) using 15 phenotypic features based on the appearance of worms (e.g., pixel intensity, area, length). Motility measurements are inferred from the magnitude of a change in a feature over time or how often its sign or direction changes (e.g., when the worm becomes longer than shorter). Two statistical methods are employed to measure significant changes in phenotypes: glass effect size (monoparametric) and Mahalanobis distance (multiparametric). The results are analyzed in “SchistoView”, a graphic interface supported by MySQL database, which allows users to visualize, query and explore NTS data. This method shows several improvements in comparison to the HCS-based assay developed by Paveley et al. (70), including a higher NTS segmentation accuracy, it requires less parasites per assay and quantifies how parasites move instead of simply determine if the movement has occurred or not. In part, these improvements were achieved due to an innovative solution that allows the segmentation of touching objects in bright-field images, a challenging task for image analysis in general. In fact, many techniques implemented in this platform, such as automated liquid handling of 100 µm-sized organisms and statistical analysis of multiparametric data, can be useful to other screening projects. The HCS approach by Chen et al. (61) was successfully used to create the phenotypic profile of known schistosomicidal agents, as well of 1,323 human approved compounds, identifying new potential drug candidates.

HCS has also been applied to screen compounds against adult forms (male and females) of schistosomes. Neves et al. (69) described a HCS-based method to extract morphological and motility measurements from time-lapse bright-field images of *S. mansoni*. In this assay, schistosomes were distributed in 96-well microplates (1 worm per well) and their images acquired over time (100 x 0.3 s interval) using a 2x objective. Image analysis was carried out in a customized pipeline of the open-source software Cellprofiler (101) comprising a series of image processing modules, including those responsible for detection of wells, illumination correction, parasites segmentation and features extraction. More than 90 features were quantified during this analysis though only two, related to worm motility, were used to describe drug-induced phenotypes. So far, this method has been applied to detect the schistomicidal effect of antidepressant paroxetine (69) as well as putative inhibitors of *S. mansoni* thioredoxin glutathione reductase (*Sm*TGR) (93, 94).

#### Non-Image-Based Methods

##### Electrical Impedance Spectroscopy

Electrical impedance spectroscopy (EIS) is noninvasive and label-free method that has been explored in schistosomiasis drug discovery (65, 71, 102, 103) (**Figure 1C**). In summary, ESI systems quantify dielectrical properties of samples while applying an alternative current (AC) electrical field using electrodes. EIS measurements can be used to detect phenotypic changes in cells/organisms induced by perturbagens, such as small molecules (104). Smout et al. (102) employed the xCELLigence real time cell analysis (RTCA) system to measure the effect of chemical compounds on the motility of helminths, including adult schistosomes. The experiments are carried out in E-plates, commercial microplates with gold electrodes embedded in the base of the wells that allow monitoring electrical resistance. Later, an improved version of this assay (xCELLigence worm real-time motility assay - xWORM) expanded its applications to detect alterations in the motility of schistosomes cercariae and egg hatching (71). xWORM is a sensitive method and was able to reveal the schistosomicidal effect of natural-derived [phytochemicals (105, 106) and puromycin (107)] and synthetic [forchlorfenuron (108) and polyridylruthenium(II) complexes (109)] compounds. Nonetheless, it is not sensitive enough to detect NTS small movements and requires a relativity larger number of samples compared to conventional microscopy (103). These limitations were addressed by Modena et al. (110) who developed a microfluid impedance-based platform to measure changes in NTS motility. Their system consisted of a microfluidic chip, made of polydimethylsiloxane (PDMS), attached to a glass substrate with patterned electrodes. This method showed high sensitivity towards both viable and non-viable parasites and required a lower number of worms per assay to operate in comparison to microscopy. Later, this concept evolved into a parallelized platform which was able to run four experiments simultaneously (103). More recently (65), the system was reformulated, becoming more automated, performing at a higher throughput (32 experiments run in parallel) and allowing long-term culturing of NTS. This assay was successful in determining the EC_50_ of mefloquine and oxethazaine which were of the same order of magnitude as those calculated by microscopy (65).

##### Isothermal Microcalorimetry

Isothermal microcalorimetry (IMC) is a very sensitive technique that measures the heat released or consumed by physical or chemical events under essentially isothermal conditions (111, 112). IMC has been used in different areas of biomedicine, such as in the detection of infection and tumors, antibiotic testing, parasitology and screening for new drugs (111, 113). Manneck et al. (64) developed an IMC-based assay to study the effect of chemical compounds on NTS and adult worms of *S. mansoni*. In this method, the overall heat production of a suspension of parasites is continuously recorded by the microcalorimeter. After the injection of a schistosomicidal compound it is expected that the heat-flow curves change their behavior indicating compounds effect on worm metabolism and/or motility (**Figure 1C**). This assay proved to be highly sensitive, capturing subtle effects that were not detected by conventional microscopy and was used to measure the schistosomicidal activity of known anti-schistosome agents (mefloquine, praziquantel) (64, 114), their isomers/racemates (115, 116), as well as mefloquine-related arylmethanols (117) and 3-alkoxy-1,2-dioxolanes (118)

#### Label-Free Methods: Concluding Remarks

In the previous topics we described the main label-free methods available today for schistosomiasis drug discovery. They represent more automated alternatives for conventional microscopy, overcoming some of its major limitations (e.g., visual phenotypic scoring). These methods vary according to several features, such as equipment/readout (e.g., microscope/image), assay cost (equipment and supplies), automation and screening throughput. They all have their pros and cons and the choice of one over another depends, in many cases, on equipment availability. In low-budget labs, video microscopy and WormAssay represent more affordable solutions for compound screening against schistosomula and/or adult schistosomes, since they require low-cost equipment, and the assays are carried out in regular microplates. On the other hand, HCS-based assays demand a high initial investment but use regular microplates as supplies, operate at a higher throughput and can be readily incorporated into automated platforms. Moreover, HCS systems can be easily coupled with a wide range of objective lens, allowing them to capture images of schistosomula, adults and potentially other parasites forms (e.g., eggs and juveniles). In contrast to image-based, non-image-based methods are less employed in screening campaigns. Overall, they demand expensive (microcalorimeter and RTCA systems) or customized (EIM platform) equipment, more costly supplies (e.g., E-plates), operate at a lower-throughput in comparison with automated microscopy and, in the case of xWORM, it is not able to detect phenotypic changes in schistosomula. Nonetheless, they are highly sensitive, may reveal drug-induced phenotypes which cannot be captured by image-based methods (e.g., metabolic activity), and xWORM already offers protocols for measuring the effect of compounds on schistosome eggs and cercariae. In conclusion, it is our understanding that HCS-based assays represent today the most advanced approaches to schistosomes phenotypic screening due to their ability to describe complex phenotypes of different forms of the parasite at a high throughput.

## Target-Based Screening

Target-based drug discovery (TDD) consists of finding ligands for a known biological target, previously identified as having potential relevance in a disease. One of its main advantages is the possibility of knowing characteristics of the target binding site, which allows the optimization of ligands and the development of an efficient structure-activity relationship. Its emergence was made possible by advances in molecular biology and genetics, which allowed the identification of individual biological targets, as well as the possibility of developing compounds that interact with these targets. The genome project, the development of techniques such as RNA interference and gene knockout, advances in structural biology and the development of computational tools were of great importance for the emergence of this alternative to the phenotypic approach in drug discovery (47, 119–121).

Ligand-binding assays are at the core of TDD strategies. In the context of pharmacological screening, the classical assay provides, for example, affinity, potency, and maximum response data of the analyzed molecules. It is also possible to determine the intrinsic activity of ligands (122, 123), through functional binding assays, in addition to assessing the residence time of the ligand to its target molecule (124). In contrast to the classical binding assays, HTS is a strategy that allows for the testing of tens of thousands of compounds per day, for activity against biological targets. Considered one of the most used strategies in TDD, HTS can be performed using different approaches, that can be mainly divided into biochemical assays and cell-based assays (125–127).

Although in-solution assays are commonly used for *in vitro* screenings, immobilized enzyme reactors (IMER) systems have proved to be a valid alternative drug screening strategy. The immobilization of a target protein to a solid support has the advantage of longer maintenance of the stability of the molecule, and the possibility of extracting the protein from the reaction medium, allowing it to be reused. In addition, IMER can be coupled to different separation systems, such as high performance liquid chromatography (HPLC), which solve the possible problem of product and assayed compounds fluorescing at the same wavelength, since these analytes can be separated and analyzed individually (128). Active anti-cancer compounds (129), enzyme inhibitors (130–133) and G protein-coupled receptors (GPCR) (134) binders have recently been identified using this approach.

After the sequencing and decoding of the *S. mansoni* genome, several putative drug targets were identified (**Table 3**), and studies using a the target-based approach emerged (157).

**Table 3:** Some classes of molecular targets in *Schistosoma* sp.

Target type	Family	Target protein	Number of screened compounds	Screening strategy	Species	Reference
**Enzyme**	Kinases	S*m*PLK1	49	Phenotypic assay	*S. mansoni*	(92)
		Tyrosine kinase	37	Phenotypic assay	*S. mansoni*	(49)
	Histone deacetylases	*Sm*HDAC8	18	Enzymatic and phenotypic assays	*S. mansoni*	(135)
		*Sm*Sirt2	36	Enzymatic and phenotypic assays	*S. mansoni*	(136)
	Redox metabolism	*Sm*TGR	59,360	Enzymatic and phenotypic assays	*S. mansoni*	(137)
		*Sm*TGR	119	Enzymatic and phenotypic assays	*S. mansoni*	(138)
	Lipid biosynthesis	*SjOAR*	14,400	Virtual screening, phenotypic and enzymatic assays	*S. japonicum*	(139)
	Phosphodiesterases	*Sm*PDE	265	Phenotypic assay	*S. mansoni*	(140)
		*Sm*PDE4A-D	1,085	Enzymatic and phenotypic assays	*S. mansoni*	(81)
		*Sm*PDE4A	975	Virtual screening and enzymatic assay	*S. mansoni*	(141)
	Proteases	*Sj*CL1	3	Enzymatic and phenotypic assays	*S. japonicum*	(142)
		*Sj*CL2	3	Enzymatic and phenotypic assays	*S. japonicum*	(142)
		*Sj*CL3	1	Phenotypic assay	*S. japonicum*	(143)
		*Sj*CD	7	Enzymatic assay	*S. japonicum*	(144)
		*Sm32* (Legumain, *Sm*AE)	23, 49, 31	Enzymatic assay	*S. mansoni*	(145–147)
		*Sm*CB1 (*Sm*31)	18, 68, 34, 39, 3	Enzymatic assay	*S. mansoni*	(148–152)
		*Sm*CD1	1	Enzymatic assay	*S. mansoni*	(153)
		*Sm*CL1 (*Sm*CF)	5	Enzymatic assay	*S. mansoni*	(154)
		*Sm*CL3	2	Enzymatic assay	*S. mansoni*	(155)
		*Sm*20S	3	Enzymatic and phenotypic assays	*S. mansoni*	(82)
		*Sm*POP	19	Enzymatic assay	*S. mansoni*	(156)
**Receptor**	GPCR	*Sm*.5HTR	143	Enzymatic assay and phenotypic	*S. mansoni*	(78)
		*Sm*.5HTR	~250	Enzymatic assay and phenotypic	*S. mansoni*	(140)
	TRP	*Sm*.TRPM_PZQ_	N/A	Phenotypic assays	*S. mansoni*	(58)

*S. mansoni* encodes 252 kinases, which have already been shown to have a relevant role in the biology of the parasite (158). *S. mansoni* polo-like kinase (*Sm*PLK1) is mainly expressed in reproductive organs of the adult parasite, which suggests a contribution of this enzyme to cell division. The screening of a series of analogues compounds, derived from a human PLK1 inhibitor bioactive against *S. mansoni* parasites, yielded the identification of potent compounds against schistosomula and adults (92). Buskes et al. (49) reported the optimization of a compound, analogous to the tyrosine kinase inhibitor lapatinib which had initially been identified as a potent antitrypanosomal. From this optimization, analogues were selected for a repurposing approach, and screened against *S. mansoni* parasites. As a result, several potent compounds against the adult form of the parasite were identified and considered promising leads for further assessment as antischistosomal compounds. The main drawback to exploit kinases as drug targets is the difficulty to achieve selectivity among the vastness of homologues present both in schistosomes and humans. One promising route to achieve this selectivity is exploring allosteric binding sites as alternatives to the more conserved active sites.

Targeting histone-modifying enzymes (HMEs) has been a widely explored strategy for the discovery of new drugs to treat parasitic diseases. In *S. mansoni*, two classes of histone deacetylases (HDAC and sirtuins) have been identified and are considered potential drug targets for the treatment of schistosomiasis (159). Kalinin et al. (135) designed and synthesized a series of compounds, derived from weak human HDAC8 (hsHDAC8) inhibitors, which varied in the size and flexibility of their side chains. These molecules were screened on *S. mansoni* HDAC8 (*Sm*HDAC8) to assess their ability to inhibit enzyme activity, and a potent and selective SmHDAC8 inhibitor was identified. Crystallographic and docking studies with *Sm*HDAC8 and the compound revealed key interactions between them, which are not observed with the human orthologue hsHDAC8. Another study identified the first *S. mansoni* sirtuin 2 (*Sm*Sirt2) inhibitors with activity in the low micromolar range, potency against larval schistosome and adult worms, and no toxicity to human cells. These inhibitors were previously identified by an *in vitro* screening of a compound library, comprising potent and specific growth inhibitors of other parasites, such as *Leishmania donovani* and *Trypanosoma cruzi* (136).

Another drug target for schistosomiasis is thioredoxin glutathione reductase (TGR), an enzyme responsible for maintaining the redox homeostasis. A high-throughput screening against a compound library comprising 59,360 synthetic compounds was carried out, of which 74 inhibited *Sm*TGR activity by more than 90% at 10 µM. Some of these had potent schistosomicidal activity against the larvae and adult worms (137). After the flood of date coming from the large q-HTS campaign, there was a feeling of certain disappointment since no major pre-clinical or clinical candidate arose from all this effort. However, the work with *Sm*TGR as a drug target is slowly picking up pace again. A recent work selected the most active chemotypes from HTS plus analogues and re-tested against the enzyme. Ninety-seven had *Sm*TGR inhibitory activity confirmed, and five of them killed *S. japonicum*, *S. haematobium* and *S. mansoni* (with LD_50_ ≤ 10 µM) adult worms, and all other development stages of *S. mansoni* (138). *Sm*TGR has also been recently explored under the fragment-based drug discovery paradigm, as it will be discussed further ahead in this review.

3-oxoacyl-ACP reductase (OAR) is an enzyme involved in lipid biosynthesis that is absent in mammals. The cloning, expression, and purification of *Schistosoma japonicum* OAR (*Sj*OAR) was performed by Liu et al. (139), as well as the elaboration of a homology model of the three-dimensional structure of this protein. A library consisting of more than 14,000 small molecules was chosen for an *in silico* screening against the model of *Sj*OAR, and 30 initial hits were identified. Of these hits, two were shown to have schistosomicidal activity on both juvenile and adult forms, relatively low cytotoxicity, and could significantly inhibit the activity of the purified recombinant enzyme, confirming that *Sj*OAR is the primary target of these compounds.

From a library focused on exploring phosphodiesterases (PDEs) as potential drug targets for several parasites, 265 compounds were obtained and had their antischistosomal activity evaluated (160). *In vivo* screening revealed that 171 of the compounds had activity against adult parasites. All these hits showed some level of activity in a mouse model, and two of them, when combined with PZQ, managed to a near complete eradication of viable eggs. Despite being structurally related to PDE10 inhibitors, further studies are needed to validate *Sm*PDEs as the targets of these compounds (140). Another important work was carried out by Long and colleagues (81), which undertook considerable efforts to validate *S. mansoni* PDE4A as a target for a series of benzoxaboroles. From a library of 1085 benzoxaboroles, the authors identified some compounds which induced hypermotility and degeneration of *S. mansoni* worms. Employing phenotypic assays with transgenic *C. elegans*, chemical and functional characterization, it was possible to observe a positive correlation between the hypermotile phenotype of the parasite and the inhibition of *Sm*PDE4A, suggesting that this enzyme is a target for the tested bezaxoboroles. In another recent study, inhibitors of *Sm*PDE4A were discovered, using a virtual screening approach. Homology models of the enzyme structure were generated and used to screen a chemical library. 25 hits were selected and tested as inhibitors of the recombinant *Sm*PDE4A, and five of them were able to inhibit its activity (141).

Schistosome aspartic proteases, as well as cysteine proteases, play a major role in life cycle of *Schistosoma* parasites by breaking down host hemoglobin an essential source of amino acids from the parasite. Studies have shown that reductions in transcript levels of *Sm*CD1, an enzyme of *S. mansoni* similar to cathepsin D, lead to phenotypic changes in the parasite, such as growth retardation (161). Thus, *Sm*CD1, as well as the orthologue from *S. japonicum*i (*Sj*CD1), are considered validated targets in antischistosomal drug discovery. A homology modelling study and SAR analysis with peptidomimetic compounds designed against *Sj*CD1 revealed unique structural features for achieving selectivity to this enzyme (144). Recombinant *Sm*CD1 was recently expressed in HEK293 cells, characterized biophysically and biochemically (153). This is an important step towards further exploring this enzyme in TDD, since they can be considered promising druggable targets as demonstrated in the past with the development of HIV-1 protease inhibitors.

With over 10 years of publications, cysteine proteases are some of the oldest targets against schistosomiasis and *S. mansoni* cysteine protease cathepsin B1 (*Sm*CB1) is one example. The *Sm*CB1 has stood out as an important target for drug development. Some studies have described structural and functional characteristics (152, 162) of how *Sm*CB1 is inhibited. Some of these applied scoring methods based on quantum mechanics (QM) to describe important interactions between vinyl sulfone chemotype inhibitors and *Sm*CB1 (163). Furthermore, these inhibitors were important to map druggable hot spots in *Sm*CB1 (150). The vinyl sulfones inhibitors also showed desirable properties such as activity in phenotypic assays, selectivity for *Sm*CB1 over human cathepsin B and metabolic stability. Besides this new *Sm*CB1 inhibitor class, in a recent publication Jiková et al. (149) showed that azanitriles chemotypes can act as potent covalent inhibitors of *Sm*CB1. Using recombinantly expressed *Sm*CB1, crystal structure determination, QM methods, phenotypic and target-based assays, these authors were able to identify azanitriles with nanomolar range potency. These studies trace an important path to the identification of new molecules with therapeutic potential to treat schistosomiasis, whilst reinforcing the importance of *Sm*CB1 as a valuable *S. mansoni* drug target.

In addition to enzymes, receptors (164–166) and transporters (62, 166) are also targeted in schistosomiasis drug discovery. Serotonin (5-HT) GPCRs have already been identified in *S. mansoni* and related to worm movement regulation (66). Marchant et al. (78) characterized the pharmacological profile of the schistosome receptor *Sm*.5HTR, a GPCR involved in worm movement, and a screening of 143 previously studied compounds was performed, leading to the identification of scaffolds that regulate the activity of this receptor Similarly, a commercial GPCR compound library has been screened against *Sm*.5HTR, and 23 compounds identified as potential antagonists, with the majority showing selective inhibition of the parasite serotonin receptor (167).

In 2019, a paper published by Park et al. (58) presented important insights regarding the role of praziquantel on schistosome worms. This work showed that PZQ activates a *S. mansoni* transient receptor potential channel (*Sm*TRPM) showing properties consistent with the observed responses on worms, like nanomolar sensitivity to PZQ, stereoselectivity and sustained Ca^2+^ entry response. The authors were able to identify nanomolar sensitive of *Sm*TRPM to (R)-PZQ isomer (eutomer), which is approximately 50 times more sensitive to (S)-PZQ. Further screening campaigns will be necessary to assess the therapeutic potential of this target. Nevertheless, these findings elevate the *Sm*TRPM as a promising clinical target to treat schistosomiasis.

### Fragment-Based Drug Discovery (FBDD)

In the last decades, fragment-based drug discovery (FBDD) has been established as an efficient approach for the identification of new biologically active compounds (168–172). To date, four marketed drugs have been discovered by FBDD (173), including vemurafenib (174), venetoclax (175), erdafitinib (160), and pexidartinib (176), while over 40 fragment-based drug candidates are in different stages of clinical trials (177). In FBDD campaigns, small and less complex compounds, commonly with molecular weight (MW) <300 Da and <20 heavy atoms, are screened against therapeutic targets (178, 179). The use of very small molecules offers advantages over screening larger compounds, including a more efficient sampling of chemical space with fewer compounds (180), higher hit rates (181), and also better physicochemical properties (182, 183). Besides, lower investments are needed, and FBDD projects progress relatively faster between the research and development (R&D) phases (184). As an example, vemurafenib took only six years from hit identification to the approval by the US Food and Drug Association (FDA) in 2011 (171, 174). Therefore, incorporating FBBD into anti-schistosome drug discovery may help to accelerate the identification and development of drug candidates for schistosomiasis and other NTDs (185, 186). FBDD involves steps of library design, screening, and optimization and these steps are discussed in the subsequent sections.

#### Fragment Library Design

Most early fragment libraries were designed based on the Rule of Three (RO3), i.e. MW ≤300 Da, the number of hydrogen bond donors ≤3, the number of hydrogen bond acceptors is ≤3 and cLogP is ≤3 (178). However, this paradigm has been changing based on incremental experience in FBDD acquired in the last years and considering the facilitation of fragment screening and/or subsequent fragment optimization chemistry (187, 188). Nowadays, several strategies exist, which cover the use of labeled fragments for nuclear magnetic resonance (NMR) spectroscopy, covalent linkage for mass spectrometry, dynamic combinatorial chemistry, X‐ray crystallographic screening of specialized fragments and fragments optimized for easy elaboration (189).

The last two strategies are blended and available to the community through the XChem fragment screening facility at Diamond Light Source in the UK (190). For this facility, a library of chemical compounds poised for expansion called DSPL (191) was designed to allow rapid and low cost follow-up synthesis and to provide quick SAR data through X-ray crystallography. Poised fragments contain at least one functional group which can be synthesized using a robust, well-characterized reaction. Reactions include amide couplings, Suzuki-type aryl-aryl couplings and reductive aminations, amongst others. The library was designed by analyzing all commercially available fragment space (using the ZINC reference library), yielding nearly 30,000 compounds of which a chemically diverse subset of 800 compounds was selected for the poised library. In practice, the available chemical material shows bias towards the most commonly used chemical reactions (192), however, it still the case that the XChem program delivers between 2% and 10% fragment hit rates (soaks yielding bound fragments in the structure) for projects amenable to multi-crystal soaking and screening by crystallography. A remarkable result exemplified by the more than 100 projects screened since 2016, including the successful screening and follow-up of the SARS-CoV-2 M^pro^ protein, also released as an open science public service (193, 194).

#### Fragment Screening Strategies

Screening strategies rely on identifying and ranking chemical fragments that bind to the protein target. Methods need to be sufficiently sensitive to measure low affinity interactions and therefore do not typically rely on activity assays. The NMR, surface plasmon resonance (SPR), thermal shift assays (TSA) (also known as differential scanning fluorimetry (DSF) and X-ray crystallography are the most widely used techniques for high throughput fragment screening.

##### Nuclear Magnetic Resonance

The NMR spectroscopy is the most robust fragment screening method for detecting very weak binding (K_D_s in the µM to mM range). The NMR approach used to identify target-ligand interactions can be based on observation of the target (target-observed) or ligand (ligand-observed) (195). In the case of ligand-observed NMR methods, only the resonance of the nuclei present in the ligands is measured (196). This type of approach includes methods such as saturation-transfer difference (STD), water LOGSY, cross saturation (CS) and transferred-cross saturation (TCS), transferred nuclear Overhauser effect (trNOE), NOE editing/filtering diffusion editing, relaxation editing, use of paramagnetic tags and residual dipolar couplings (197, 198). On the other hand, the target-observed methods provide data on the target nuclei that are directly involved in the interaction with the ligand (197). Among the target-observed methods, there are chemical shift mapping using ^15^N-HSQC, backbone amide hydrogen exchange and solvent paramagnetic relaxation enhancement methods. In comparison with other methods, NMR spectroscopy has the advantage of being conducted in solution, which allows the protein to be as close as possible to its native conformation. In addition, NMR enables both the target and the ligand to be structurally characterized serving as quality control assay to verify the structural integrity of the ligand. However, compared to other methods, fragment screening by NMR is relatively slow (199).

##### Surface Plasmon Resonance

Screening by SPR involves immobilization of the target protein on a gold or silver sensor surface and measurement in the change in reflected light following ligand interaction (200, 201). The method is high throughput and very sensitive (µM to nM range) providing kinetic binding data (K_on_ and K_off_ rates), from which, K_D_s are calculated. The main disadvantage of the technique is the potential difficulty of immobilizing proteins in native conformation and therefore it is important to test a reference compound to assess correct binding behavior. The relatively high concentration of immobilized target and fragment affinities can lead to false positives through non-specific binding (202).

##### Thermal Shift Assays

In the thermal shift assay protein denaturation is monitored by fluorescence either intrinsic tryptophan fluorescence or using dyes that preferentially bind partially or completely denatured proteins (203). Ligand binding is measured indirectly as the increase in thermal stability resulting from interaction with the target protein in the native state (204). This method is easy, fast and inexpensive for fragment screening (185). However, could not be appropriate for all target proteins, because indirect readout of the protein’s denaturation, and chances to generate false positives. Thereby, it is necessary to confirm the identified hits with other methods (205).

##### X-ray Crystallography

Crystallography is the current method for delivering atomic resolution information and is arguably the method of choice for primary screening if a project is amenable to this approach, i.e., access to high quality purified protein that can be reproducibly crystallized (206). There are two strategies for obtaining protein-fragment complexes, namely, co-crystallization or soaking (207). Co-crystallization is the mixing of the free protein in solution with a ligand prior to crystallization, which allows the small molecule to bind to the protein prior to crystal lattice formation. This is the preferred method if a protein complex with a specific ligand is required. A potential downside is co-crystallization with different small molecules can lower the success rate for crystallization, or introduce changes in resolution and crystal form, burdening the downstream analysis and limiting high throughput. In crystal soaking, false negatives come from protein failing to crystallize, or protein crystals growing without bound compounds. A simpler approach is soaking, where the compound is added, (dissolved or pure), directly to the crystallization drop which already contains crystals. In this method, compounds diffuse through solvent channels in the crystal accessing binding pockets in the protein (208). However, false negatives can still arise through a lack of fragment binding. Compounds may also dissolve crystals by disrupting the crystal lattice. Both co-crystallization and soaking are sensitive to low compound solubility.

#### Fragment-to-Lead (F2L) Optimization

**Figure 2:**
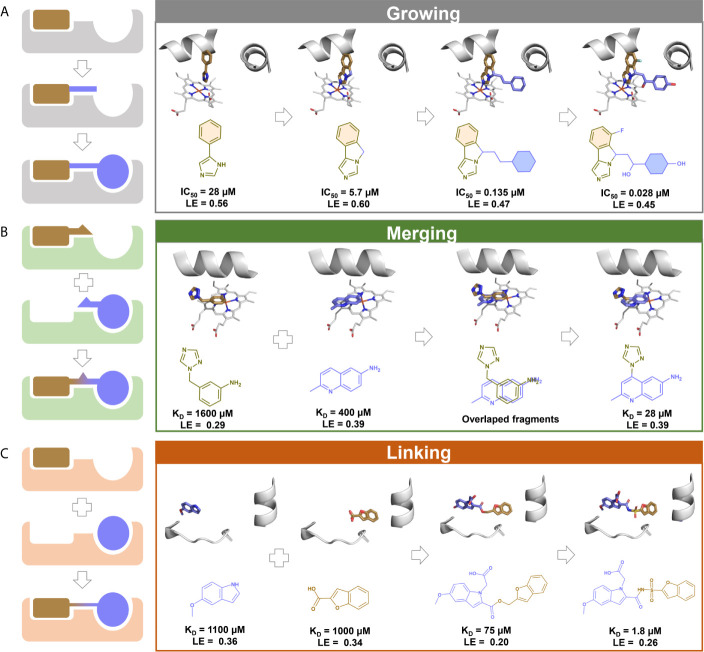
A schematic illustration of fragment optimization strategies. **(A)** Fragment growing: initial fragment with low affinity is optimized by stepwise addition of functional groups to obtain a larger compound with high affinity. 3D and 2D schemes represents the growing evolution of navoximod, an indoleamine 2,3-deoxygenase 1 (IDO1) inhibitor with antineoplastic properties (solid tumors) (215); **(B)** Fragment merging: two or more fragments sharing the same pocket are covalently merged to obtain a larger compound with higher affinity. 3D and 2D schemes represent an example of fragment merging to the discovery of inhibitors of the *Mycobacterium tuberculosis* cytochrome P450 CYP121 (216). **(C)** Fragment linking: two or more fragments bound independently in proximity are covalently linked with suitable linkers to obtain a larger compound with higher affinity. 3D and 2D schemes represent an example of fragment linking to the discovery of inhibitors of *M. tuberculosis* pantothenate synthetase (217).

The fragment hits commonly have weak binding affinities (from mM to high µM range) as a consequence of the reduced number of heavy atoms to form attractive interactions with the target (209). Despite the low MW, the fragment hits form high-quality interactions, i.e., highly energetically favorable interactions that surpass the entropic penalties for binding (210, 211). Thus, fragments constitute starting points that can be optimized iteratively into larger higher-affinity compounds – a process known as fragment-to-lead (F2L) (205, 210). The F2L process is guided by the information of binding mode, growth vectors available, and ligand efficiency (LE) and its derivatives (212, 213). LE is a metric used to describe the average free energy of binding per heavy atom (Equation 1) (214), and LE ≥0.3 is frequently used to select the most promising fragment hits to F2L (183). Three main strategies are used to optimize fragments, including fragment growing, merging, and linking (**Figure 2**).

$LE=\frac{\Delta G}{HAC}$ (1) where, ΔG is the free energy of binding and HAC is the heavy atoms count of a compound.

Fragment growing (**Figure 2A**) is the most commonly applied strategy in F2L (218). The effectiveness of this strategy is shown by its use in F2L process of three out of the four marketed drugs derived from FBDD (173), namely vemurafenib (174), erdafitinib (160), and pexidartinib (176). The fragment growing strategy involves a several steps. Firstly, potential growth vectors are identified in the chemical structure of the fragment hit (212, 219). Then, atoms or chemical groups are added to the fragment hit to explore additional interactions with the binding site and increase the potency (205). Structural information of the binding mode is essential during fragment growing to identify potential sub-pockets to explore and assess the maintenance of the fragment’s original binding mode and additional molecular interactions (198, 220). At each iteration of growing, synthesis and testing, success can be evaluated by LE, monitoring if the extra molecular mass added was beneficial (221).

Fragment merging (**Figure 2B**) can be applied when two fragments bind in an overlapping position of the binding site and can be merged into a unique and more potent hybrid compound (222, 223). As in fragment linking, both fragments can work additively when merged or even synergistically (224). Here, structural information is also crucial to understand the binding mode (198). Fragment merging is also difficult to achieve and less frequently used because of the challenging task of maintaining the original binding modes of the fragments after merge (225).

Fragment linking consists of the connection of fragments binding to different but adjacent sub-pockets in the binding site by a linker moiety (**Figure 2C**) (168, 177) and is the most powerful strategy for converting fragments into potent ligands (198). This is due to the potential super additivity effect, where the binding free energy of linked fragments is higher than the sum of binding free energy of the individual fragments (226, 227). The main challenge is the design of a linker group that does not affect the original binding mode of fragment hits (218). As a successful case, the marketed drug venetoclax was optimized by applying the fragment linking method (173, 177).

The *Sm*TGR is a flavoenzyme expressed by schistosomes involved in the detoxification pathways that are pivotal for their survival in the host organism (228–230). Most *Sm*TGR inhibitors are reactive electrophilic compounds, such as metal derivatives or Michael acceptors, presumably targeting the nucleophilic residues (selenocysteine and low pKa redox active cysteines), which may result in low selectivity and toxicity (231, 232). Attempts to obtain crystal structures of *Sm*TGR in complex with such inhibitors have been unsuccessful, reflecting the problem of crystallizing non-homogenous protein preparations resulting from the presence of several redox and nucleophilic centers in the *Sm*TGR, which are the sites of action for electrophilic inhibitor (233).

**Figure 3:**
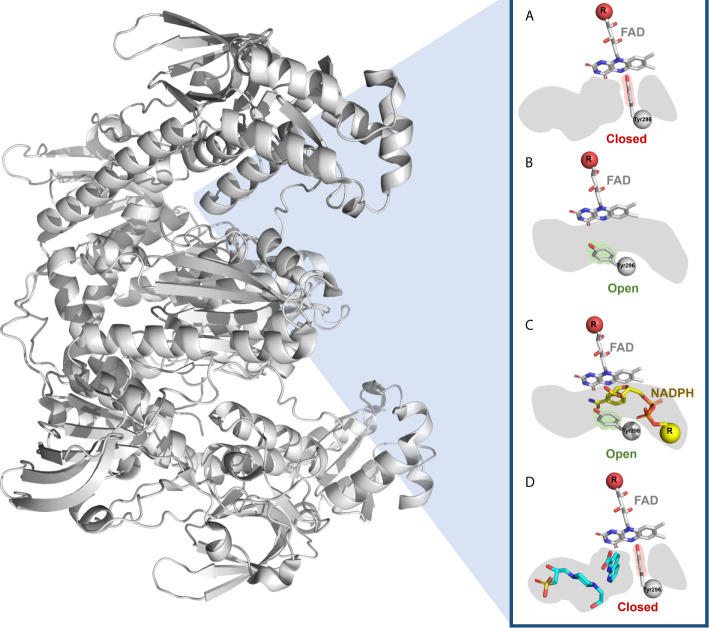
Doorstop pocket of *Sm*TGR adjacent to the NADPH binding site. The Tyr296 of the doorstop pocket is represented in **(A)** closed and **(B)** open conformation, as well as in the presence of **(C)** NADPH and **(D)** two fragments (1,8-naphthyridine-2-carboxylate and 1-(2-hydroxyethyl)piperazine).

Given the challenges posed by the redox properties of the enzyme, allosteric and secondary binding sites could be explored, as they present less reactive amino acids which could lead to less toxic and more selective inhibitors. For this reason, Silvestri and coworkers (233) prioritized 1,000 fragment inhibitors of the *Sm*TGR from a quantitative HTS campaign. Then, by X-ray crystallography identified two fragments (1,8-naphthyridine-2-carboxylate and 1-(2-hydroxyethyl)piperazine) that bound in a secondary pocket adjacent to the NADPH binding site (**Figure 3**), named as “doorstop pocket”. The pockets are separated by the Tyr296 residue, where the aromatic ring of Tyr296 could adopt the closed (**Figures 3A, D**) and open (**Figures 3B, C**) conformations. Small molecules bound at the doorstop pocket disturb the well-known and conserved conformational adjustments associated with NADPH binding and enzyme reduction (233). Subsequently, chimeric compounds blending the structural features of the initial fragments into single compounds were synthesized and showed improved *Sm*TGR inhibition activity, *ex vivo* activity against larval and adult *S. mansoni* worms at low micromolar concentrations. In addition, the designed compounds tended to have selectivity for *Sm*TGR, as the amino acid residues of the doorstop pocket are not conserved between members of the FAD/NAD-linked reductase family (233). Although strictly this work was not a FBDD campaign (at least not originally designed as one), it was an interesting effort that showed the potential of the fragment-based approach to disclose new binding sites that can be explored to develop novel potent ligands.

Although FBDD has been established as an efficient approach for the identification of new biologically active compounds for several diseases, very few applications had been reported for schistosomiasis. Thus, FBDD has a great potential for anti-schistosomal drug discovery in the future.

#### *In Silico* Approaches for FBDD

*In silico* approaches have been used in several parts of FBDD pipelines as an alternative or complementary approach, with the benefits of speed and low cost (234, 235). Many fragment libraries are available in the literature (188) and computational methods can be used to design a fragment library with high chemical diversity, synthetically accessible to be easily optimized during F2L, and also select or exclude fragments based on physicochemical properties (236, 237). The biophysical techniques applied for fragment screening are low-to-medium throughput (188), limiting the number of fragments that can practically be screened, and therefore the coverage of chemical space (180, 238). To compensate for this, molecular docking and machine learning can be used to virtually screen a large number of fragments and prioritize the most promising for experimental testing (224, 239–242).

Several *in silico* methods are also used during the F2L process (243). When no structural information about the binding is available, molecular docking and molecular dynamics are used to predict the binding mode and inform the growing, linking, and merging strategies (244, 245). The fragment hits can also be optimized with the help of *de novo*, machine learning, and deep learning methods (209, 243). These methods will be discussed in more details in the next sections.

## Computer Assisted- and Artificial Intelligence-Based Drug Design

### Schistosome Post-Genomic Era

#### Genes and Proteins Functional Annotation

The “-omics” era for schistosomiasis drug discovery started when the first versions of the S. mansoni (157, 246), S. haematobium (247), and S. japonicum (248) genomes were published. Recently, revised versions of the S. japonicum (249) and S. haematobium (250) genomes were released, enhancing the quality of the available genomics data for these three main trematode responsible for the majority of schistosomiasis cases in the world. The latest genome versions of these trematode species, together with other parasitic worms species, are available online at (https://parasite.wormbase.org/species.html#Platyhelminthes).

Most of the genome of schistosomes (like many other organisms) has yet to be explored experimentally. Consequently, bioinformatic tools and resources have become pivotal for the functional annotation and analysis of genes and their products. There are a wide range of general resources available that host biological information (genomics sequences, transcription data, protein structures, metabolomic data and more, see **Table 4**) as well as bioinformatic tools to perform analysis to unveil useful information within the data. Databases and webservers such as (but not limited to) InterPro (251), CATH-Gene3D (252, 253), the Conserved Domains Database (CDD) (254), HAMAP (196), PANTHER (255), Pfam (256, 257), PROSITE Patterns and Profiles (258), ProDom (196), PIRSF (196), PRINTS (259), SMART (260), Structure-Function Linkage Database (SFLD) (261), SUPERFAMILY (262, 263), TIGRFAMs (264) are integrated to identify specific motifs and domains to classify the protein of interest. Other resources such as the Kyoto Encyclopedia of Genes and Genomes (KEGG) database (265) provide functional annotation that encompasses molecular-level information about biological systems, integrating molecular datasets resulting from genome sequencing or other large-scale experimental technology. The KEGG website (https://www.genome.jp/kegg/) offers several tools to find data-oriented and organism-specific entry points, as well as analytical tools for diverse ends, such as genome and metagenome functional annotation (BlastKOALA and GhostKOALA respectively), pathway mapping tools (KEGG Mapper), sequence and chemical similarity search (BLAST/FASTA and SIMCOMP respectively).

**Table 4:** Databases and webservers for gene and protein functional annotation.

Resource	Link	Reference
CATH-Gene3D	http://www.cathdb.info/browse/sunburst?from_cath_id=1.10	(252, 253)
CDD	https://www.ncbi.nlm.nih.gov/cdd/	(254)
GO	http://www.geneontology.org	(266)
HAMAP	https://hamap.expasy.org/	(196)
InterPro	https://www.ebi.ac.uk/interpro/	(251)
KEGG database	https://www.genome.jp/kegg/	(265)
PANTHER	http://pantherdb.org/	(255)
Pfam	http://pfam.xfam.org/	(256, 257)
PIRSF	https://proteininformationresource.org/pirwww/dbinfo/pirsf.shtml	(196)
PRINTS	http://130.88.97.239/PRINTS/index.php	(259)
ProDom	http://prodom.prabi.fr/prodom/current/html/home.php	(196)
PROSITE	https://prosite.expasy.org/	(258)
SFLD database	http://sfld.rbvi.ucsf.edu/archive/django/index.html	(261)
SMART	http://smart.embl-heidelberg.de/	(260)
SUPERFAMILY	https://supfam.org/	(262, 263)
TIGRFAM	http://tigrfams.jcvi.org/cgi-bin/index.cgi	(264)
WormBase	https://parasite.wormbase.org	(268)

Gene Ontology (GO) model (266) (http://www.geneontology.org) is commonly used for describing genes using a unified and common vocabulary applicable to any organism. GO is a hierarchical way of describing information gathered on genes and proteins at different levels of annotation and is used by many of the databases mentioned above as it provides the top three different categories of high-quality annotation: (*i*) biological process, (*ii*) molecular function, and (*iii*) cellular component; each one referring to the biological objective of the gene/gene product, biochemical activity of the gene/gene product, and place in the cell where the gene product is active respectively (267). The unified annotation/vocabulary provided by GO is dynamic and entirely based on the principle of shared orthology by all eukaryotic organisms. It can be updated as the ontologies become mature through the integration of more experimental results.

As an exemplar of how these tools and resources can be used in the context of *Schistosoma* is by Padalino and colleagues (40) who identified *S. mansoni* Lysine Specific Demethylase-1 (*Sm*LSD1) as a druggable epigenetic target, as well as daunorubicin and pirarubicin as potential inhibitors. This was possible using bioinformatics tools, such as Uniprot, PROSITE, InterPro, and Pfam, BLAST in combination with homology modeling, molecular docking, and a whole-organism screening.

Likewise, the latest revised versions of *S. haematobium* and *S. japonicum* published by Stroehlein et al. (250) and Luo et al. (249) demonstrate the use of several tools in an extensive way. Luo and colleagues (249) combine tools whose functions range from genome evaluation to RNA, protein prediction and phylogenetic analysis and all of them converge to the evaluation and comparison of the revised genome and the previously published versions. Stroehlein and colleagues (250) used a lesser extent of tools, however a deep comparison between previously published versions of *Schistosoma* genomes was conducted and a careful data curation was carried out to ensure the quality of the assembled genome.

Since 2017, many efforts in terms of RNA-seq data have been reported in the context of Schistosoma (85, 269–272). A thorough protocol of how to gather, process, reconstruct the transcripts, and identify novel long non-coding RNA (lncRNAs), as well as their expression levels (273). In this protocol, Maciel and Verjovski-Almeida use a set of open-source Unix-based tools combined with several R (274) packages to support the analysis of differential expression of some lncRNAs. Wang and colleagues (85) reported a large RNAi screening against S. mansoni to uncover new therapeutic targets. The authors conducted a GO enrichment to better understand the roles of the essential genes to the parasite development and attachment to substrate. Furthermore, this study demonstrated the essentiality of SmTK25 kinase to maintain the muscular function of the parasite, thus representing a promising therapeutic target.

#### Phylogenetic Analysis – Computational Phylogenomic Inference Methods

One of the principal methods for integrating and inferring functional annotation is phylogenetics - the study of the evolutionary story of organisms and their relationships with other organisms or group of organisms. The relationships among the organisms are described in a detailed and hierarchical manner through phylogenomic inference methods, which will result in a phylogeny, represented by a phylogenetic tree (275). It is the best way for identifying and confirming whether two or more sequences are orthologs (276).

Phylogenomic inference methods are applied to assign a biological function to an unannotated gene or protein (277). Their overall accuracy is high and theoretically the topology of a generated phylogenetic tree is correct unless highly dissimilar sequences (identity <25%) are present among the aligned sequences (278). The methods are directly dependent on a multiple sequence alignment (MSA), whereby main objective is to align more than two sequences, allowing the identification of conserved motifs, domains and regions in the compared sequences (nucleic or amino acids) (277). Therefore, the quality of a final phylogenetic tree will strongly depend on the MSA quality and accuracy. The most popular tools for phylogenetic analysis are PhyML (279), RAxML/ExaML (280), FastTree (281), and IQ-TREE (281, 282).

There are many methods that can be used to build phylogenetic trees from an MSA. Distance-based methods such as neighbor-joining and Unweighted Pair Group Method with Arithmetic Mean (UPGMA) are the simplest examples which provide a genetic distance calculation between the multiple sequences aligned, but do not give evolutionary information (275, 282, 283). More complex methods, such as maximum parsimony, minimum evolution, and maximum likelihood can be employed considering the Bayes’ theorem for the estimation of an evolutionary model (284, 285).

Maximum parsimony’s principle relies on the sum of the number of minimum possible sites substitutions in each sequence. The sum will constitute the tree length for the investigated topology and the topology with the minimum length is called maximum parsimony tree (279). Minimum evolution is a distance-based method which generates a tree topology based on the lowest value among the values obtained from the sum of all branches (286). This method has a high time-cost mainly when dealing with too many sequences, e.g. protein superfamilies. The Maximum likelihood statistical method is known as the method which produces the most reliable phylogenetic trees in comparison with distance-based methods and the parsimony method (283). A phylogenetic tree is constructed through the maximum likelihood method accordingly to the following steps: (*i*) generate a starting tree; (*ii*) rearrange the starting tree through topological substitutions and evaluate the new tree; (*iii*) replace the starting tree and repeat the step *ii* if no better tree is identified; on the contrary, terminate the search (283).

For the cited methods, even for those based on distance, the robustness of the tree is assured by a bootstrap resampling technique (275, 282, 287) which is based on the replacement of nucleotides, codons or amino acids and the construction of a new tree with the new sequences. Next, each interior branch of the original tree is compared to the newly branches and, if the branches are different, a bootstrap score 0 is given while a score 1 is assigned to the other branches. The process is repeated a few hundred times, the percentage of times which the bootstrap score 1 was given is calculated, and the topology can be considered correct if the percentage is equal or greater than 95%. In the context of *Schistosoma* species and other helminths, these types of phylogenetic analysis have been boosted by the availability of a wide range of high quality genomes captured and analyzed within the 50 Helminth Genome Project (https://www.sanger.ac.uk/collaboration/50hgp/) and well as the other genomes and resources found in WormBase (268).

### Cheminformatics

Despite all advances achieved in the field of automation of screens, FBDD, and also in the understanding of disease biology in the post-genomic era, delivering new drugs to the market remains a highly complex, expensive and time-consuming process (288, 289). Therefore, there is a need for innovative approaches that could bring new drugs for patients at a lower cost-to-market. In this context, computer-assisted drug design approaches (CADD) has been considered as a potential opportunity (290, 291). Cheminformatics is a field of CADD and has the objective of utilizing computer and information sciences to solve problems in the area of chemistry (292, 293). This involves the design, creation, retrieval, storage, management, organization, analysis, visualization, dissemination, and use of chemical information (294). Over the last few years, the advances in data processing power and the development of new artificial intelligence (AI) tools, has fueled the field of CADD and cheminformatics (295, 296). Moreover, AI tools abilities have increasingly been applied to a wide variety of chemical challenges, from improving computational chemistry to end-to-end drug discovery as well as to synthesis planning/prediction (297, 298).

The developments in phenotypic and target-based screening provide data essential for applying computational tools to accelerate the discovery of new drugs to treat schistosomiasis (299, 300). These advances coupled with data storage in public databases such as PubChem (301–303) and ChEMBL (304) have enabled the compilation, curation, analysis, and application of chemical and biological information to support antischistosomal lead generation and optimization (305–307). Thus, cheminformatics has an important role in schistosomiasis drug discovery through the conversion of data to information and information into knowledge (308, 309), supporting data-driven decisions in lead identification and optimization (310, 311).

In the last years, pivotal advances in cheminformatics-driven drug discovery have been achieved in three main sub-fields: molecular *de novo* design, virtual screening, and synthesis prediction. Machine learning approaches have also progressively been applied in these areas. Therefore, in the next sections, machine learning approaches will be described and important aspects to schistosomiasis drug discovery will be highlighted.

#### Machine Learning

Machine learning (ML), mainly supervised methods, is a growing field of AI that uses different algorithms to enable computers to learn from sample data, known as “training data”, without being explicitly programmed for this task (312). ML algorithms are capable of recognizing complex patterns in chemical structures that evade human rationales because of the enormous number of parallel variables that should be addressed in drug design (313). On the other hand, molecular modeling techniques (e.g., docking, molecular dynamics) are based on explicit physical equations derived from molecular mechanics and quantum mechanics theory (314). Consequently, ML techniques are considered to have higher predictive value than classic molecular modeling methods. Combining human and ML -derived models should enable medicinal chemists to make better decisions and move projects forward more quickly (315, 316).

ML has applications in several stages of drug discovery and development, accelerating the overall process (317), including automation of whole-organism assays (70, 94), lead identification and optimization (318), and clinical development, for example in patient recruitment, prediction of diagnosis, prognosis, treatment planning, and clinical trial outcomes (317, 319). ML provides robust methods such as random forest (RF) (320, 321) for learning from large and multi-dimensional chemical data to make predictions and select new chemical entities for experimental testing (322). The generation of ML models for drug design and discovery consists of a multi-step protocol (323). The first step is the data collection of chemical and biological information from the literature and/or databases, followed by preparation and curation of data employing standardized protocols (324–326). Then, descriptors are calculated from molecular representations varying from one-dimension to *n*-dimensions (327). These molecular descriptors are derived from a logical and mathematical method that converts the chemical information into a useful number (328). The third step is the model training (learning), where a ML technique is applied to establish Quantitative Structure-Activity Relationships (QSAR) between the molecular descriptors and continuous (e.g., pIC_50_, K_i_, etc.) or categorical/binary (e.g., active, inactive, toxic, nontoxic, etc.) experimental bioactivities or properties (329, 330). The models that are developed need to be validated using appropriate metrics to assess their predictive value (331, 332), and then used to predict the biological activity of new compounds (318).

It is worth pointing out that the initial training data underpins ML models generation. The data should be high-quality and in sufficient quantity to lead in models with high performance (295). However, in the current scenario, the data of pharmaceutical industry is scarce, costly, and need substantial resources, which could limit the use of ML for drug discovery (329).

Some guidelines for model generation and validation should be followed to ensure the reliability of the model. In this context, some principles for assessing the validity of ML-based QSARs have been proposed by the Organization for Economic Cooperation and Development (OECD) (333) stating that they should have:

- **A defined endpoint:** Ensure clarity in the endpoint being predicted by a given model, since biological property could be determined by different protocols and under different experimental conditions;
- **An unambiguous algorithm:** ensure reproducibility in the ML algorithm that generates predictions of an endpoint from chemical structure.
- **A defined applicability domain (AD):** the AD is defined as the chemical space containing the features of the compounds used to train the ML-based QSAR models (334). The AD offers means to assess the confidence of prediction to unseen compounds (335). The most common methods to define AD use distance-based metrics to calculate the distance of the features between the training set and a new compound being predicted (335–337).
- **Appropriate measures of goodness-of-fit, robustness, and predictivity:** ensure the distinction between the internal performance of a model (as represented by goodness-of-fit and robustness) and the predictivity of a model (as determined by external validation);
- **Mechanistic interpretation, if possible**: ensure that some consideration is given to the possibility of a mechanistic association between the descriptors used in a model and the endpoint being predicted (333).

As an example of application of ML to schistosomiasis drug discovery, Zorn and coworkers (338) used data from phenotypic screens against the schistosomula and adult stages of *S. mansoni* to develop ML models. Firstly, the authors elaborated two rule books and associated scoring systems used to normalize 3,898 phenotypic data points and transform to categorical data. Then, using the Assay Central software, they generated eight Bayesian machine learning models based on each developmental stage of the parasite and four experimental time points (≤24, 48, 72, and >72 h). Subsequently, the generated models were used to predict the activity of compounds from several libraries of commercial vendors. Finally, 40 compounds predicted as active and 16 compounds predicted as inactive were selected and purchased for *in vitro* phenotypic assays against schistosomula and adult stages of *S. mansoni*. In this manner, the authors achieved a prediction accuracy for active and inactives of 61% and 56% for schistosomula and adults, respectively. Additionally, the hit rates achieved were 48% and 34% for schistosomula and adults, respectively (338).

#### Deep Learning

DL is a type of ML that uses a hierarchical recombination of features to extract pertinent information and then learn the patterns represented in the data. In other words, DL uses artificial neural networks (ANNs) with many layers of nonlinear processing units for learning data representations. DL has emerged to deal with the high volume and exponentially growth of sparse data, coming from different sources around the globe (339). Conceptually, DL was conceived in the 1980s, with the development of ANNs, which, at the time, could not out-perform ML algorithms due to the small amounts of data available. As soon as advances in hardware were achieved, in 2010s, with graphic processing units and cloud computing technologies, deep neural networks (DNNs) became more popular and able to be trained and accomplish complex tasks (340).

**Figure 4:**
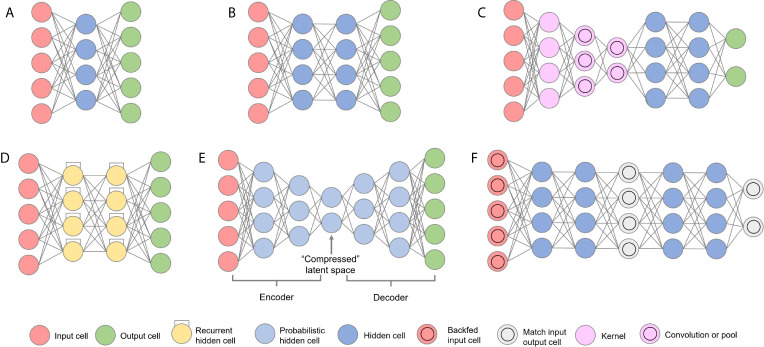
Architecture of several popular neural networks. **(A)** classical Feed Forward Network; **(B)** Deep Feed Forward Network; **(C)** Convolutional Neural Network; **(D)** Recurrent Neural Network; **(E)** Variational Autoencoder Network; and **(F)** Generative Adversarial Network.

The basic structure of a classical ANN and DL representations are represented in **Figure 4** and are inspired by the structure of the human brain. There are three basic layers in a neural network: the input layer, hidden layer and output layer. Depending on the type of ANN, the nodes, also called neurons, in neighboring layers are either fully connected or partially connected. The major difference between DL and traditional ANN is the complexity of the NNs. Traditional ANNs (**Figure 4A**) normally only have one hidden layer whereas DL architectures such as Deep Feed Forward Network (**Figure 4B**) uses larger numbers of hidden layers.

Since the advent of QSAR in the 1960s for drug discovery projects, and the use of so called “shallow methods” for the identification of new chemical entities with drug like properties (341), the application of DL methods has been increasing (276, 315, 317–323). The applications of DL can be as diverse as the creativity of those who applies and develops the methods. The possibilities are unlimited in terms of algorithms [see (342)] but are restricted in terms of data quality and chemical space coverage (325). However, DL has broadened even the ability of generating new chemical data, allowing the usage of autoencoders to interpret SMILES data and, within that chemical space, generate new scaffolds sharing a few physicochemical properties with their parental molecules (343). From virtual screening processes to synthesis prediction, DL has been largely used in the field of CADD, and its applications are exemplified in the next sections.

##### Artificial Intelligence-Assisted Virtual Screening (VS)

As a fundamental part of CADD strategies, virtual screening (VS) is an *in silico* screening alternative to the experimental HTS approach to search libraries of small molecules and identify those structures which are most likely to have biological activity (344, 345). VS represents a rapid and low-cost and method for screen promising compounds against pathogens, cells and/or specific biological targets (344, 346). A VS campaign is basically a funnel-like process (347) composed of different filters. A large chemical database can be submitted to those different filters and, throughout the process, the compounds presenting undesired properties will be filtered out. In the end of the process, virtual hits with drug-, lead- or even fragment-like properties are presented in a ranked list.

In the last few years, the success of machine and deep learning has enabled the development of VS methods that can extract task-specific features directly from chemical data (295, 296). Convolutional Neural Networks (CNN, **Figure 4C**) are a subclass of DL that search for recurring spatial patterns in data and compose them into complex features in a hierarchical manner (348, 349). Chemical descriptors have very high dimensionality, and hence training a standard Feedforward network to recognize chemical patterns would require hundreds of thousands of input cells. This can cause many problems associated with the “curse of dimensionality” in neural networks. The CNNs provide a solution to this (350) by utilizing convolutional and pooling layers to help reduce the dimensionality from compound graphs (351). As convolutional layers are trainable but have significantly fewer parameters than a standard hidden layer, they can highlight important parts of the chemical structure and pass each of them forward.

Although DL have important advantages, the most prominent demonstration of DL’s capability are in areas where large amount of data are available, which is not the reality of all drug discovery campaigns (297, 352). In addition, studies demonstrated that simpler ML methods can outperform DL for activity prediction (353).

##### Deep Generative Models

Regardless of the advances in cheminformatics, the conception of the large majority of new molecules in drug discovery campaigns comes from the inventiveness of medicinal chemists (354). Since the 1990s, *de novo* methods have been used to design new molecules from scratch, commonly using structure-based approaches and resulting in compounds that are sterically and electrostatically complementary to the binding site of a protein target (355). However, the molecules generated by early *de novo* design methods were usually synthetically challenging, with poor pharmacokinetic properties, and the generation process required long runtimes (356, 357).

With the progress in deep learning, a variation of the *de novo* design method called generative modeling has appeared as a promising approach (358). These methods model the underlying probability distribution of chemical features from a training dataset and, thus, learn the essential aspects that characterize molecules (296, 359). Then, new molecules are generated combining these features by sampling the learned distribution of chemical features (360). The most common deep learning architectures for generative models are Recurrent neural networks (RNNs) (361), generative adversary network (GAN) (362), and variational autoencoder (VAE) (**Figure 4**) (363).

The RNNs (**Figure 4D**) are commonly trained with a large number of Simplified Molecular Input Line-Entry System (SMILES) strings, which encode chemical structures (364). Then, the RNN predict the probability of the next SMILES character considering a sequence of preceding characters (365). Thereby, the new molecules are generated by RNN character by character until the required number of characters have been produced (360).

The VAEs (**Figure 4E**) are composed by an autoencoder model that contains an encoder and a decoder network. The encoder translate a higher-dimensional molecular representation (e.g., SMILES) into a lower-dimensional representation, called latent space (366). The decoder translate the latent-space representation back to the higher-dimensional representation to generate new molecules (295, 366, 367). In addition, this network uses probabilistic hidden cells, which applies a radial basis function to the difference between the test sample and the cells’ mean. In this sense, VAE learns the parameters of a probability distribution representing the chemical structure data. Instead of just learning a function representing the chemical space, it gains a more detailed and nuanced view of the chemical structures, sampling from the distribution and generating new chemical structures (359).

GANs (**Figure 4F**) consist of two specialized networks that “contest” with each other: a generative network and a discriminative network (367). With careful regulation, these two adversaries compete with each other, each’s drive to succeed improving the other. The end result is a well-trained generator that can spit out a new chemical structure with desired biological property. The generative network (usually a CNN) tries to generates new molecules, while discriminative network tries to discern generated molecules as artificial or real (296). Mechanistically, discriminating network receives either training data or generated content from the generative network. How well the discriminating network was able to correctly predict the biological property is then used as part of the error for the generating network. Both networks are trained alternatively aiming the generation of molecules that are indiscernible from the real data (319).

In addition to the deep learning architectures for generative modeling, it is possible to use techniques such as transfer learning and reinforcement learning to fine-tune the models to generate molecules with the desired properties (e.g., activity against a target and physicochemical properties) and also optimize compounds such as fragments (368, 369). The power of these methodologies is the design of new molecules with ideal properties in shorter periods and lower costs (370, 371).

Despite the innovation of generative models, the novelty and accessibility of generated molecules must be evaluated (372–374). Gao and Coley (375) observed that generative models can produce infeasible molecules even with good performance in benchmarks. On the other hand, in a work for the discovery of discoidin domain receptor family member 1 (DDR1) kinase inhibitors (370), Walters and Murcko (366) pointed out that the top inhibitor is very similar to a known DDR1 inhibitor (366, 371).

#### Synthesis Prediction

The synthetic feasibility of virtual compounds identified in VS campaigns is a key point when considering synthesizing and further optimizing their properties (376, 377). Efforts from several research groups to improve the evaluation of synthetic routes and their inherent accessibility have been published and well-known software has been produced, e.g. SYNCHEM (378), RASA (379), LHASA (376), CAMEO (380), SOPHIA (381), EROS (382), and Reaxys (295, 383). Their main goal is to assess synthetically accessible routes, reaction predictions, and start material selection. To achieve this goal, approaches based on basic rules for organic synthesis, data-driven intelligent systems, sequence-to-sequence, template-based models, knowledge-graph based, and retrosynthetic prediction models have been proposed and published (384–386). To list the main obstacles for reaching a good accuracy in predicting both accessible synthetic routes and retrosynthetic disconnections, we can point out (*i*) the low number of unsuccessful reactions reported, (*ii*) the extensive data curation process, which impacts on the data quality and, consequently the predicted outcomes (384, 385). The current state-of-the art relies on treating the task as a text processing problem. Natural Language Processing (NLP) algorithms have been tested, implemented, and shown to provide of promising outcomes. The IBM RXN platform (387) represents a successful application and example of how to deal with chemical reactions as text. The platform uses the simplified molecular-input line-entry system (SMILES) as the source of local and global features potentially involved in a chemical reaction (388).

As well as in FBDD, very few applications of cheminformatics have been reported for schistosomiasis. Thus, cheminformatics also has a great potential for anti-schistosomal drug discovery in the future.

## Concluding Remarks and Future Directions

In conclusion, we would like to emphasize that the recent advances in automation of whole organism screening and target-based assays, as well as FBDD, CADD and AI tools integrated in drug design projects, represent a new era in anti-schistosomiasis drug discovery. The phenotypic assay methods described here are more sensitive and faster than traditional microscopy an have enabled the identification of several new antischistosomal candidates. In parallel, significant contributions are coming from the genomics to target-based screening approaches, especially with prospecting and prioritizing biological targets with key/essential roles in parasite survival and/or host-parasite interactions. The automated collection and processing of X-ray crystallography data at synchrotons has transformed fragment-based screening enabling the acquisition of structural data at atomic resolution. The generation of large datasets from advances in the automation of phenotypic screening and target-based approaches has created a fertile ground for drug discovery. These data have enabled the use of artificial intelligence tools, such as machine learning and deep learning, to generate predictive QSAR models for prioritization of VS hits or structural design of novel compounds. These tools can be also used for *in silico* multi-parameter optimization, for achieving a favorable balance between target potency, selectivity, physicochemical, pharmacokinetic and toxicological properties. Therefore, we see the use of AI tools and QSAR models as a time-, labor-, and cost-effective way to discover hit compounds and to optimize lead candidates in the early stages of drug discovery process. We hope that these new technologies collectively will empower schistosomiasis drug discovery and increase the efficiency of the various processes involved to deliver new drugs to the market.

## Author Contributions

All authors contributed to the article and approved the submitted version.

## Funding

The authors would like to thank Brazilian funding agencies, CNPq, CAPES, FAPERJ, FAPEG and FIOCRUZ for financial support and fellowships. JM-F, AS, and LS are supported by CAPES fellowship (Finance code 001). CA and FS-J are CNPq research fellows. AS thanks CAPES for PDSE fellowship to perform part of his Ph.D. studies in UK (#88881.131626/2016-01). NF is supported by the Medical Research Council (Grant No: MR/T000171/1). 

## Conflict of Interest

The authors declare that the research was conducted in the absence of any commercial or financial relationships that could be construed as a potential conflict of interest.

The reviewer CC declared a past co-authorship with one of the authors CA to the handling Editor.

## References

[1] Gryseels B Polman K Clerinx J Kestens L. Human Schistosomiasis. Lancet (2006) 368:1106–18. 10.1016/S0140-6736(06)69440-3 16997665

[2] WHO. Schistosomiasis. In: Schistosomiasis (2021). Available at: https://www.who.int/en/news-room/fact-sheets/detail/schistosomiasis (Accessed May 5, 2021).

[3] Amoah AS Hoekstra PT Casacuberta-Partal M Coffeng LE Corstjens PLAM Greco B. Sensitive Diagnostic Tools and Targeted Drug Administration Strategies are Needed to Eliminate Schistosomiasis. Lancet Infect Dis (2020) 20:e165–72. 10.1016/S1473-3099(20)30254-1 32595046

[4] Hotez PJ Alvarado M Basáñez MG Bolliger I Bourne R Boussinesq M. The Global Burden of Disease Study 2010: Interpretation and Implications for the Neglected Tropical Diseases. PloS Negl Trop Dis (2014) 8:e2865. 10.1371/journal.pntd.0002865 25058013 PMC4109880

[5] Kassebaum NJ Arora M Barber RM Bhutta ZA Brown J Carter A. Global, Regional, and National Disability-Adjusted Life-Years (DALYs) for 315 Diseases and Injuries and Healthy Life Expectancy (HALE), 1990–2015: A Systematic Analysis for the Global Burden of Disease Study 2015. Lancet (2016) 388:1603–58. 10.1016/S0140-6736(16)31460-X PMC5388857 27733283

[6] Colley DG Bustinduy AL Secor WE King CH. Human Schistosomiasis. Lancet (2014) 383:2253–64. 10.1016/S0140-6736(13)61949-2 PMC4672382 24698483

[7] Nation CS Da’dara AA Marchant JK Skelly PJ. Schistosome Migration in the Definitive Host. PloS Negl Trop Dis (2020) 14:1–12. 10.1371/journal.pntd.0007951 PMC7117656 32240157

[8] McManus DP Dunne DW Sacko M Utzinger J Vennervald BJ Zhou X-N. Schistosomiasis. Nat Rev Dis Primers (2018) 4:13. 10.1038/s41572-018-0013-8 30093684

[9] Castillo MG Humphries JE Mourão MM Marquez J Gonzalez A Montelongo CE. Biomphalaria Glabrata Immunity: Post-genome Advances. Dev Comp Immunol (2020) 104:103557. 10.1016/j.dci.2019.103557 31759924 PMC8995041

[10] Eyayu T Zeleke AJ Worku L. Current Status and Future Prospects of Protein Vaccine Candidates Against Schistosoma Mansoni Infection. Parasite Epidemiol Control (2020) 11:e00176. 10.1016/j.parepi.2020.e00176 32923703 PMC7475110

[11] Molehin AJ. Schistosomiasis Vaccine Development: Update on Human Clinical Trials. J BioMed Sci (2020) 27:1–7. 10.1186/s12929-020-0621-y 31969170 PMC6977295

[12] Cioli D Pica-Mattoccia L Basso A Guidi A. Schistosomiasis Control: Praziquantel Forever? Mol Biochem Parasitol (2014) 195:23–9. 10.1016/j.molbiopara.2014.06.002 24955523

[13] Mäder P Rennar GA Ventura AMP Grevelding CG Schlitzer M. Chemotherapy for Fighting Schistosomiasis: Past, Present and Future. ChemMedChem (2018) 13:2374–89. 10.1002/cmdc.201800572 30212614

[14] Gönnert R Andrews P. Praziquantel, a New Broad-Spectrum Antischistosomal Agent. Z Parasitenkd (1977) 52:129–50. 10.1007/BF00389899 410178

[15] Zwang J Olliaro P. Efficacy and Safety of Praziquantel 40 mg/kg in Preschool-Aged and School-Aged Children: A Meta-Analysis. Parasit Vectors (2017) 10:47. 10.1186/s13071-016-1958-7 28126024 PMC5270314

[16] Secor WE Montgomery SP. Something Old, Something New: Is Praziquantel Enough for Schistosomiasis Control? Future Med Chem (2015) 7:681–4. 10.4155/fmc.15.9 PMC4795816 25996059

[17] Caffrey CR. Chemotherapy of Schistosomiasis: Present and Future. Curr Opin Chem Biol (2007) 11:433–9. 10.1016/j.cbpa.2007.05.031 17652008

[18] Olliaro P Delgado-Romero P Keiser J. The Little We Know About the Pharmacokinetics and Pharmacodynamics of Praziquantel (Racemate and R-Enantiomer). J Antimicrob Chemother (2014) 69:863–70. 10.1093/jac/dkt491 24390933

[19] Meyer T Sekljic H Fuchs S Bothe H Schollmeyer D Miculka C. Taste, A New Incentive to Switch to (R)-Praziquantel in Schistosomiasis Treatment. PloS Negl Trop Dis (2009) 3:e357. 10.1371/journal.pntd.0000357 19159015 PMC2614124

[20] Wang W Wang L Liang Y-S. Susceptibility or Resistance of Praziquantel in Human Schistosomiasis: A Review. Parasitol Res (2012) 111:1871–7. 10.1007/s00436-012-3151-z 23052781

[21] Fallon PG Doenhoff MJ. Drug-Resistant Schistosomiasis: Resistance to Praziquantel and Oxamniquine Induced in Schistosoma Mansoni in Mice is Drug Specific. Am J Trop Med Hyg (1994) 51:83–8. 10.4269/ajtmh.1994.51.83 8059919

[22] Ismail MM Taha SA Farghaly AM El-Azony AS. Laboratory Induced Resistance to Praziquantel in Experimental Schistosomiasis. J Egypt Soc Parasitol (1994) 24:685–95. 7844435

[23] Couto FF Coelho PMZ Araújo N Kusel JR Katz N Jannotti-Passos LK. Schistosoma Mansoni: A Method for Inducing Resistance to Praziquantel Using Infected Biomphalaria Glabrata Snails. Mem Inst Oswaldo Cruz (2011) 106:153–7. 10.1590/S0074-02762011000200006 21537673

[24] Fallon PG Sturrock RF Niang AC Doenhoff MJ. Short Report: Diminished Susceptibility to Praziquantel in a Senegal Isolate of Schistosoma Mansoni. Am J Trop Med Hyg (1995) 53:61–2. 10.4269/ajtmh.1995.53.61 7625534

[25] Ismail M Bennett JL Tao L-F Farghaly A Bruce J Metwally A. Characterization of Isolates of Schistosoma Mansoni From Egyptian Villagers That Tolerate High Doses of Praziquantel. Am J Trop Med Hyg (1996) 55:214–8. 10.4269/ajtmh.1996.55.214 8780463

[26] Crellen T Walker M Lamberton PHL Kabatereine NB Tukahebwa EM Cotton JA. Reduced Efficacy of Praziquantel Against Schistosoma Mansoni Is Associated With Multiple Rounds of Mass Drug Administration. Clin Inf Dis (2016) 63:1151–9. 10.1093/cid/ciw506 PMC5064161 27470241

[27] Kabuyaya M Chimbari MJ Manyangadze T Mukaratirwa S. Efficacy of Praziquantel on Schistosoma Haematobium and Re-Infection Rates Among School-Going Children in the Ndumo Area of uMkhanyakude District, KwaZulu-Natal, South Africa. Infect Dis Poverty (2017) 6:1–9. 10.1186/s40249-017-0293-3 28385154 PMC5383960

[28] Melman SD Steinauer ML Cunningham C Kubatko LS Mwangi IN Wynn NB. Reduced Susceptibility to Praziquantel Among Naturally Occurring Kenyan Isolates of Schistosoma Mansoni. PloS Negl Trop Dis (2009) 3:e504. 10.1371/journal.pntd.0000504 19688043 PMC2721635

[29] Ramirez B Bickle Q Yousif F Fakorede F Mouries M-A Nwaka S. Schistosomes: Challenges in Compound Screening. Expert Opin Drug Discov (2007) 2:S53–61. 10.1517/17460441.2.S1.S53 23489033

[30] Katsuno K Burrows JN Duncan K van Huijsduijnen RH Kaneko T Kita K. Hit and Lead Criteria in Drug Discovery for Infectious Diseases of the Developing World. Nat Rev Drug Discov (2015) 14:751–8. 10.1038/nrd4683 26435527

[31] Caffrey CR Secor WE. Schistosomiasis: From Drug Deployment to Drug Development. Curr Opin Infect Dis (2011) 24:410–7. 10.1097/QCO.0b013e328349156f 21734570

[32] Spangenberg T. Alternatives to Praziquantel for the Prevention and Control of Schistosomiasis. ACS Infect Dis (2020). 10.1021/acsinfecdis.0c00542 32819092

[33] Geary TG Sakanari JA Caffrey CR. Anthelmintic Drug Discovery: Into the Future. J Parasitol (2015) 101:125–33. 10.1645/14-703.1 25584662

[34] Lombardo FC Pasche V Panic G Endriss Y Keiser J. Life Cycle Maintenance and Drug-Sensitivity Assays for Early Drug Discovery in Schistosoma Mansoni. Nat Protoc (2019) 14:461–81. 10.1038/s41596-018-0101-y 30610241

[35] Tekwu EM Anyan WK Boamah D Baffour-Awuah KO Tekwu SK Beng VP. Mechanically Produced Schistosomula as a Higher-Throughput Tools for Phenotypic Pre-Screening in Drug Sensitivity Assays: Current Research and Future Trends. Biomark Res (2016) 4:21. 10.1186/s40364-016-0075-2 27895916 PMC5120492

[36] Pica-Mattoccia L Cioli D. Sex- and Stage-Related Sensitivity of Schistosoma Mansoni to In Vivo and In Vitro Praziquantel Treatment. Int J Parasitol (2004) 34:527–33. 10.1016/j.ijpara.2003.12.003 15013742

[37] Tavares NC de Aguiar PHN Gava SG Oliveira G Mourão MM. Schistosomiasis: Setting Routes for Drug Discovery. In: Special Topics in Drug Discovery. London, UK: InTech (2016). p. 116–24. 10.5772/65386

[38] Lalli C Guidi A Gennari N Altamura S Bresciani A Ruberti G. Development and Validation of a Luminescence-based, Medium-Throughput Assay for Drug Screening in Schistosoma Mansoni. PloS Negl Trop Dis (2015) 9:e0003484. 10.1371/journal.pntd.0003484 25635836 PMC4312041

[39] Panic G Flores D Ingram-Sieber K Keiser J. Fluorescence/Luminescence-Based Markers for the Assessment of Schistosoma Mansoni Schistosomula Drug Assays. Parasit Vectors (2015) 8:624. 10.1186/s13071-015-1233-3 26644133 PMC4672532

[40] Padalino G Ferla S Brancale A Chalmers IW Hoffmann KF. Combining Bioinformatics, Cheminformatics, Functional Genomics and Whole Organism Approaches for Identifying Epigenetic Drug Targets in Schistosoma Mansoni. Int J Parasitol Drugs Drug Resist (2018) 8:559–70. 10.1016/j.ijpddr.2018.10.005 PMC6288008 30455056

[41] Keiser J. In Vitro and In Vivo Trematode Models for Chemotherapeutic Studies. Parasitology (2010) 137:589–603. 10.1017/S0031182009991739 19961653

[42] Mansour NR Bickle QD. Comparison of Microscopy and Alamar Blue Reduction in a Larval Based Assay for Schistosome Drug Screening. PloS Negl Trop Dis (2010) 4:e795. 10.1371/journal.pntd.0000795 20706580 PMC2919390

[43] Panic G Vargas M Scandale I Keiser J. Activity Profile of an FDA-Approved Compound Library Against Schistosoma Mansoni. PloS Negl Trop Dis (2015) 9:e0003962. 10.1371/journal.pntd.0003962 26230921 PMC4521867

[44] Maccesi M Aguiar PHN Pasche V Padilla M Suzuki BM Montefusco S. Multi-Center Screening of the Pathogen Box Collection for Schistosomiasis Drug Discovery. Parasit Vectors (2019) 12:1–10. 10.1186/s13071-019-3747-6 31640761 PMC6805474

[45] Zheng W Thorne N McKew JC. Phenotypic Screens as a Renewed Approach for Drug Discovery. Drug Discov Today (2013) 18:1067–73. 10.1016/j.drudis.2013.07.001 PMC4531371 23850704

[46] Ban TA. The Role of Serendipity in Drug Discovery. Dialogues Clin Neurosci (2006) 8:335–44. 10.31887/dcns.2006.8.3/tban PMC3181823 17117615

[47] Croston GE. The Utility of Target-Based Discovery. Expert Opin Drug Discov (2017) 12:427–9. 10.1080/17460441.2017.1308351 28306350

[48] Moffat JG Vincent F Lee JA Eder J Prunotto M. Opportunities and Challenges in Phenotypic Drug Discovery: An Industry Perspective. Nat Rev Drug Discov (2017) 16:531–43. 10.1038/nrd.2017.111 28685762

[49] Buskes MJ Clements M Bachovchin KA Jalani HB Leonard A Bag S. Structure–Bioactivity Relationships of Lapatinib Derived Analogs Against Schistosoma Mansoni. ACS Med Chem Lett (2020) 11:258–65. 10.1021/acsmedchemlett.9b00455 PMC7073886 32184954

[50] Miraj CMAG Fan WH Song LJ Yu CX Feng B. Synthesis and SAR Studies of New Oxadiazole-2-Oxide Derivatives With Remarkable In Vitro Activity Against Schistosoma Japonicum. J Microb Biochem Technol (2017) 09:535–43. 10.4172/1948-5948.1000339

[51] Wu J Wang C Leas D Vargas M White KL Shackleford DM. Progress in Antischistosomal N,N′-diaryl Urea SAR. Bioorg Med Chem Lett (2018) 28:244–8. 10.1016/j.bmcl.2017.12.064 PMC6026081 29317164

[52] Aulner N Danckaert A Ihm JE Shum D Shorte SL. Next-Generation Phenotypic Screening in Early Drug Discovery for Infectious Diseases. Trends Parasitol (2019) 35:559–70. 10.1016/j.pt.2019.05.004 31176583

[53] Swinney DC Lee JA. Recent Advances in Phenotypic Drug Discovery. F1000Research (2020) 9:944. 10.12688/f1000research.25813.1 PMC7431967 32850117

[54] Peak E Chalmers IW Hoffmann KF. Development and Validation of a Quantitative, High-Throughput, Fluorescent-Based Bioassay to Detect Schistosoma Viability. PloS Negl Trop Dis (2010) 4:e759. 10.1371/journal.pntd.0000759 20668553 PMC2910722

[55] Abdulla M-H Ruelas DS Wolff B Snedecor J Lim K-C Xu F. Drug Discovery for Schistosomiasis: Hit and Lead Compounds Identified in a Library of Known Drugs by Medium-Throughput Phenotypic Screening. PloS Negl Trop Dis (2009) 3:e478. 10.1371/journal.pntd.0000478 19597541 PMC2702839

[56] Braun L Hazell L Webb AJ Allan F Emery AM Templeton MR. Determining the Viability of Schistosoma Mansoni Cercariae Using Fluorescence Assays: An Application for Water Treatment. PloS Negl Trop Dis (2020) 14:e0008176. 10.1371/journal.pntd.0008176 32214320 PMC7138324

[57] Guidi A Lalli C Gimmelli R Nizi E Andreini M Gennari N. Discovery by Organism Based High-Throughput Screening of New Multi-Stage Compounds Affecting Schistosoma Mansoni Viability, Egg Formation and Production. PloS Negl Trop Dis (2017) 11:e0005994. 10.1371/journal.pntd.0005994 28985236 PMC5646872

[58] Park SK Gunaratne GS Chulkov EG Moehring F McCusker P Dosa PI. The Anthelmintic Drug Praziquantel Activates a Schistosome Transient Receptor Potential Channel. J Biol Chem (2019) 294:18873–80. 10.1074/jbc.AC119.011093 PMC6901322 31653697

[59] Aguiar PHN Fernandes NMGS Zani CL Mourão MM. A High-Throughput Colorimetric Assay for Detection of Schistosoma Mansoni Viability Based on the Tetrazolium Salt XTT. Parasit Vectors (2017) 10:300. 10.1186/s13071-017-2240-3 28637488 PMC5480175

[60] Marcellino C Gut J Lim KC Singh R McKerrow J Sakanari J. WormAssay: A Novel Computer Application for Whole-Plate Motion-Based Screening of Macroscopic Parasites. PloS Negl Trop Dis (2012) 6:e1494. 10.1371/journal.pntd.0001494 22303493 PMC3269415

[61] Chen S Suzuki BM Dohrmann J Singh R Arkin MR Caffrey CR. A Multi-Dimensional, Time-Lapse, High Content Screening Platform Applied to Schistosomiasis Drug Discovery. Commun Biol (2020) 3:747. 10.1038/s42003-020-01402-5 33349640 PMC7752906

[62] El-shehabi F Taman A Moali LS El-sakkary N Ribeiro P. A Novel G Protein-Coupled Receptor of Schistosoma Mansoni (SmGPR-3) Is Activated by Dopamine and Is Widely Expressed in the Nervous System. PloS Negl Trop Dis (2012) 6:e1523. 10.1371/journal.pntd.0001523 22389736 PMC3289605

[63] Lee H Moody-Davis A Saha U Suzuki BM Asarnow D Chen S. Quantification and Clustering of Phenotypic Screening Data Using Time-Series Analysis for Chemotherapy of Schistosomiasis. BMC Genomics (2012) 13:1–24. 10.1186/1471-2164-13-S1-S4 22369037 PMC3471343

[64] Manneck T Braissant O Haggenmuller Y Keiser J. Isothermal Microcalorimetry to Study Drugs Against Schistosoma Mansoni. J Clin Microbiol (2011) 49:1217–25. 10.1128/JCM.02382-10 PMC3122815 21270220

[65] Ravaynia PS Lombardo FC Biendl S Dupuch MA Keiser J Hierlemann A. Parallelized Impedance-Based Platform for Continuous Dose-Response Characterization of Antischistosomal Drugs. Adv Biosyst (2020) 4:1900304. 10.1002/adbi.201900304 PMC7354102 32510834

[66] Patocka N Sharma N Rashid M Ribeiro P. Serotonin Signaling in Schistosoma Mansoni: A Serotonin–Activated G Protein-Coupled Receptor Controls Parasite Movement. PloS Pathog (2014) 10:e1003878. 10.1371/journal.ppat.1003878 24453972 PMC3894222

[67] McCusker P Chan JD. Anti-Schistosomal Action of the Calcium Channel Agonist FPL-64176. Int J Parasitol Drugs Drug Resist (2019) 11:30–8. 10.1016/j.ijpddr.2019.08.006 PMC6796685 31561039

[68] Asarnow D Rojo-Arreola L Suzuki BM Caffrey CR Singh R. The QDREC Web Server: Determining Dose-Response Characteristics of Complex Macroparasites in Phenotypic Drug Screens. Bioinformatics (2015) 31:1515–8. 10.1093/bioinformatics/btu831 PMC4410654 25540182

[69] Neves BJ Dantas RF Senger MR Valente WCG Rezende-Neto J de M Chaves WT. The Antidepressant Drug Paroxetine as a New Lead Candidate in Schistosome Drug Discovery. Med Chem Commun (2016) 7:1176–82. 10.1039/C5MD00596E

[70] Paveley RA Mansour NR Hallyburton I Bleicher LS Benn AE Mikic I. Whole Organism High-Content Screening by Label-Free, Image-Based Bayesian Classification for Parasitic Diseases. PloS Negl Trop Dis (2012) 6:e1762. 10.1371/journal.pntd.0001762 22860151 PMC3409125

[71] Rinaldi G Loukas A Brindley PJ Irelan JT Smout MJ. Viability of Developmental Stages of Schistosoma Mansoni Quantified With xCELLigence Worm Real-Time Motility Assay (xWORM). Int J Parasitol Drugs Drug Resist (2015) 5:141–8. 10.1016/j.ijpddr.2015.07.002 PMC4534758 26288742

[72] McCusker P Mian MY Li G Olp MD Tiruveedhula VVNPB Rashid F. Non-Sedating Benzodiazepines Cause Paralysis and Tissue Damage in the Parasitic Blood Fluke Schistosoma Mansoni. PloS Negl Trop Dis (2019) 13:e0007826. 10.1371/journal.pntd.0007826 31730614 PMC6881066

[73] Schneider CA Rasband WS Eliceiri KW. NIH Image to ImageJ: 25 Years of Image Analysis. Nat Methods (2012) 9:671–5. 10.1038/nmeth.2089 PMC5554542 22930834

[74] El-Sakkary N Chen S Arkin MR Caffrey CR Ribeiro P. Octopamine Signaling in the Metazoan Pathogen Schistosoma Mansoni: Localization, Small-Molecule Screening and Opportunities for Drug Development. Dis Model Mech (2018) 11:dmm033563. 10.1242/dmm.033563 29925529 PMC6078403

[75] Chan JD Agbedanu PN Grab T Zamanian M Dosa PI Day TA. Ergot Alkaloids (Re)Generate New Leads as Antiparasitics. PloS Negl Trop Dis (2015) 9:e0004063. 10.1371/journal.pntd.0004063 26367744 PMC4569474

[76] Chan JD Acharya S Day TA Marchant JS. Pharmacological Profiling an Abundantly Expressed Schistosome Serotonergic GPCR Identifies Nuciferine as a Potent Antagonist. Int J Parasitol Drugs Drug Resist (2016) 6:364–70. 10.1016/j.ijpddr.2016.06.001 PMC5196489 27397763

[77] Duguet TB Glebov A Hussain A Kulkarni S Mochalkin I Geary TG. Identification of Annotated Bioactive Molecules That Impair Motility of the Blood Fluke Schistosoma Mansoni. Int J Parasitol Drugs Drug Resist (2020) 13:73–88. 10.1016/j.ijpddr.2020.05.002 32531750 PMC7284125

[78] Marchant JS Harding WW Chan JD. Structure-Activity Profiling of Alkaloid Natural Product Pharmacophores Against a Schistosoma Serotonin Receptor. Int J Parasitol Drugs Drug Resist (2018) 8:550–8. 10.1016/j.ijpddr.2018.09.001 PMC6287472 30297303

[79] Weeks JC Roberts WM Leasure C Suzuki BM Robinson KJ Currey H. Sertraline, Paroxetine, and Chlorpromazine are Rapidly Acting Anthelmintic Drugs Capable of Clinical Repurposing. Sci Rep (2018) 8:1–17. 10.1038/s41598-017-18457-w 29343694 PMC5772060

[80] Monti L Cornec A Oukoloff K Kovalevich J Prijs K Alle T. Congeners Derived From Microtubule-Active Phenylpyrimidines Produce a Potent and Long-Lasting Paralysis of Schistosoma Mansoni In Vitro. ACS Infect Dis (2020). 10.1021/acsinfecdis.0c00508 PMC8641298 33135408

[81] Long T Rojo-Arreola L Shi D El-Sakkary N Jarnagin K Rock F. Phenotypic, Chemical and Functional Characterization of Cyclic Nucleotide Phosphodiesterase 4 (PDE4) as a Potential Anthelmintic Drug Target. PloS Negl Trop Dis (2017) 11:e0005680. 10.1371/journal.pntd.0005680 28704396 PMC5526615

[82] Bibo-Verdugo B Wang SC Almaliti J Ta AP Jiang Z Wong DA. The Proteasome as a Drug Target in the Metazoan Pathogen, Schistosoma Mansoni. ACS Infect Dis (2019) 5:1802–12. 10.1021/acsinfecdis.9b00237 PMC7283364 31355632

[83] Padalino G. WormassayGP2. 10.5281/zenodo.3929417.

[84] Padalino G Chalmers IW Brancale A Hoffmann KF. Identification of 6-(piperazin-1-yl)-1,3,5-triazine as a Chemical Scaffold With Broad Anti-Schistosomal Activities. Wellcome Open Res (2020) 5:169. 10.12688/wellcomeopenres.16069.1 32904763 PMC7459852

[85] Wang J Paz C Padalino G Coghlan A Lu Z Gradinaru I. Large-Scale RNAi Screening Uncovers Therapeutic Targets in the Parasite Schistosoma Mansoni. Science (2020) 369:1649–53. 10.1126/science.abb7699 PMC7877197 32973031

[86] Padalino G Celatka CA Kalin JH Cole PA Lassalle D Chalmers IW. Schistosoma Mansoni Lysine Specific Demethylase 1 (SmLSD1 ) is a Druggable Target Involved in Parasite Survival , Oviposition and Stem Cell Proliferation. (2020). 10.1101/2020.09.17.301184

[87] Singh R Pittas M Heskia I Xu F McKerrow J Caffrey CR. Automated Image-Based Phenotypic Screening for High-Throughput Drug Discovery. In: 2009 22nd IEEE International Symposium on Computer-Based Medical Systems. New Jersey, USA: IEEE (2009). p. 1–8. 10.1109/CBMS.2009.5255338

[88] Asarnow DE Singh R. Segmenting the Etiological Agent of Schistosomiasis for High-Content Screening. IEEE Trans Med Imaging (2013) 32:1007–18. 10.1109/TMI.2013.2247412 23428618

[89] Singh R Beasley R Long T Caffrey CR. Algorithmic Mapping and Characterization of the Drug-Induced Phenotypic-Response Space of Parasites Causing Schistosomiasis. IEEE/ACM Trans Comput Biol Bioinform (2018) 15:469–81. 10.1109/TCBB.2016.2550444 PMC5915339 27071187

[90] Asarnow D Singh R. Determining Dose-Response Characteristics of Molecular Perturbations in Whole-Organism Assays Using Biological Imaging and Machine Learning. In: 2018 IEEE International Conference on Bioinformatics and Biomedicine (BIBM). New Jersey, USA: IEEE (2018). p. 283–90. 10.1109/BIBM.2018.8621083

[91] Rojo-Arreola L Long T Asarnow D Suzuki BM Singh R Caffrey CR. Chemical and Genetic Validation of the Statin Drug Target to Treat the Helminth Disease, Schistosomiasis. PloS One (2014) 9:e87594. 10.1371/journal.pone.0087594 24489942 PMC3906178

[92] Long T Neitz RJ Beasley R Kalyanaraman C Suzuki BM Jacobson MP. Structure-Bioactivity Relationship for Benzimidazole Thiophene Inhibitors of Polo-Like Kinase 1 (PLK1), a Potential Drug Target in Schistosoma Mansoni. PloS Negl Trop Dis (2016) 10:e0004356. 10.1371/journal.pntd.0004356 26751972 PMC4709140

[93] Melo-Filho CC Dantas RF Braga RC Neves BJ Senger MR Valente WCG. QSAR-Driven Discovery of Novel Chemical Scaffolds Active Against Schistosoma Mansoni. J Chem Inf Model (2016) 56:1357–72. 10.1021/acs.jcim.6b00055 PMC5283162 27253773

[94] Neves BJ Dantas RF Senger MR Melo-Filho CC Valente WCG de Almeida ACM. Discovery of New Anti-Schistosomal Hits by Integration of QSAR-Based Virtual Screening and High Content Screening. J Med Chem (2016) 59:7075–88. 10.1021/acs.jmedchem.5b02038 PMC5844225 27396732

[95] Mansour NR Paveley R Gardner JMF Bell AS Parkinson T Bickle Q. High Throughput Screening Identifies Novel Lead Compounds With Activity Against Larval, Juvenile and Adult Schistosoma Mansoni. PloS Negl Trop Dis (2016) 10:e0004659. 10.1371/journal.pntd.0004659 27128493 PMC4851381

[96] Giuliani S Silva AC Borba JVVB Ramos PIP Paveley RA Muratov EN. Computationally-Guided Drug Repurposing Enables the Discovery of Kinase Targets and Inhibitors as New Schistosomicidal Agents. PloS Comput Biol (2018) 14:e1006515. 10.1371/journal.pcbi.1006515 30346968 PMC6211772

[97] Crusco A Whiteland H Baptista R Forde-Thomas JE Beckmann M Mur LAJ. Antischistosomal Properties of Sclareol and Its Heck-Coupled Derivatives: Design, Synthesis, Biological Evaluation, and Untargeted Metabolomics. ACS Infect Dis (2019) 5:1188–99. 10.1021/acsinfecdis.9b00034 31083889

[98] Whatley KCL Padalino G Whiteland H Geyer KK Hulme BJ Chalmers IW. The Repositioning of Epigenetic Probes/Inhibitors Identifies New Anti-Schistosomal Lead Compounds and Chemotherapeutic Targets. PloS Negl Trop Dis (2019) 13:e0007693. 10.1371/journal.pntd.0007693 31730617 PMC6881072

[99] Crusco A Bordoni C Chakroborty A Whatley KCL Whiteland H Westwell AD. Design, Synthesis and Anthelmintic Activity of 7-Keto-Sempervirol Analogues. Eur J Med Chem (2018) 152:87–100. 10.1016/j.ejmech.2018.04.032 29698860

[100] Whiteland HL Chakroborty A Forde-Thomas JE Crusco A Cookson A Hollinshead J. An Abies Procera-Derived Tetracyclic Triterpene Containing a Steroid-Like Nucleus Core and a Lactone Side Chain Attenuates In Vitro Survival of Both Fasciola Hepatica and Schistosoma Mansoni. Int J Parasitol Drugs Drug Resist (2018) 8:465–74. 10.1016/j.ijpddr.2018.10.009 PMC6216039 30399512

[101] Lamprecht MR Sabatini DM Carpenter AE. CellProfiler^TM^: Free, Versatile Software for Automated Biological Image Analysis. Biotechniques (2007) 42:71–5. 10.2144/000112257 17269487

[102] Smout MJ Kotze AC McCarthy JS Loukas A. A Novel High Throughput Assay for Anthelmintic Drug Screening and Resistance Diagnosis by Real-Time Monitoring of Parasite Motility. PloS Negl Trop Dis (2010) 4:e885. 10.1371/journal.pntd.0000885 21103363 PMC2982823

[103] Chawla K Modena MM Ravaynia PS Lombardo FC Leonhardt M Panic G. Impedance-Based Microfluidic Assay for Automated Antischistosomal Drug Screening. ACS Sens (2018) 3:2613–20. 10.1021/acssensors.8b01027 PMC6396876 30426744

[104] Modena MM Chawla K Misun PM Hierlemann A. Smart Cell Culture Systems: Integration of Sensors and Actuators Into Microphysiological Systems. ACS Chem Biol (2018) 13:1767–84. 10.1021/acschembio.7b01029 PMC5959007 29381325

[105] Wangchuk P Pearson MS Giacomin PR Becker L Sotillo J Pickering D. Compounds Derived From the Bhutanese Daisy, Ajania Nubigena, Demonstrate Dual Anthelmintic Activity Against Schistosoma Mansoni and Trichuris Muris. PloS Negl Trop Dis (2016) 10:e0004908. 10.1371/journal.pntd.0004908 27490394 PMC4973903

[106] Wangchuk P Giacomin PR Pearson MS Smout MJ Loukas A. Identification of Lead Chemotherapeutic Agents From Medicinal Plants Against Blood Flukes and Whipworms. Sci Rep (2016) 6:32101. 10.1038/srep32101 27572696 PMC5004179

[107] Yan H-B Smout MJ Ju C Folley AE Skinner DE Mann VH. Developmental Sensitivity in Schistosoma Mansoni to Puromycin to Establish Drug Selection of Transgenic Schistosomes. Antimicrob Agents Chemother (2018) 62:e02568–17. 10.1128/AAC.02568-17 PMC6105839 29760143

[108] Zeraik AE Galkin VE Rinaldi G Garratt RC Smout MJ Loukas A. Reversible Paralysis of Schistosoma Mansoni by Forchlorfenuron, a Phenylurea Cytokinin That Affects Septins. Int J Parasitol (2014) 44:523–31. 10.1016/j.ijpara.2014.03.010 PMC4071124 24768753

[109] Sundaraneedi MK Tedla BA Eichenberger RM Becker L Pickering D Smout MJ. Polypyridylruthenium(II) Complexes Exert Anti-Schistosome Activity and Inhibit Parasite Acetylcholinesterases. PloS Negl Trop Dis (2017) 11:e0006134. 10.1371/journal.pntd.0006134 29240773 PMC5746282

[110] Modena MM Chawla K Lombardo F Burgel SC Panic G Keiser J. Impedance-Based Detection of Schistosoma Mansoni Larvae Viability for Drug Screening. In: 2017 IEEE Biomedical Circuits and Systems Conference (BioCAS). New Jersey, USA: IEEE (2017). p. 1–4. 10.1109/BIOCAS.2017.8325227 PMC7116545 33409508

[111] Braissant O Keiser J Meister I Bachmann A Wirz D Göpfert B. Isothermal Microcalorimetry Accurately Detects Bacteria, Tumorous Microtissues, and Parasitic Worms in a Label-Free Well-Plate Assay. Biotechnol J (2015) 10:460–8. 10.1002/biot.201400494 PMC4406140 25511812

[112] Braissant O Bachmann A Bonkat G. Microcalorimetric Assays for Measuring Cell Growth and Metabolic Activity: Methodology and Applications. Methods (2015) 76:27–34. 10.1016/j.ymeth.2014.10.009 25461776

[113] Braissant O Wirz D Göpfert B Daniels AU. Biomedical Use of Isothermal Microcalorimeters. Sensors (2010) 10:9369–83. 10.3390/s101009369 PMC3230962 22163413

[114] Manneck T Keiser J Müller J. Mefloquine Interferes With Glycolysis in Schistosomula of Schistosoma Mansoni Via Inhibition of Enolase. Parasitology (2012) 139:497–505. 10.1017/S0031182011002204 22309769

[115] Manneck T Braissant O Ellis W Keiser J. Schistosoma Mansoni: Antischistosomal Activity of the Four Optical Isomers and the Two Racemates of Mefloquine on Schistosomula and Adult Worms In Vitro and In Vivo. Exp Parasitol (2011) 127:260–9. 10.1016/j.exppara.2010.08.011 20732321

[116] Meister I Ingram-Sieber K Cowan N Todd M Robertson MN Meli C. Activity of Praziquantel Enantiomers and Main Metabolites Against Schistosoma Mansoni. Antimicrob Agents Chemother (2014) 58:5466–72. 10.1128/AAC.02741-14 PMC4135865 24982093

[117] Ingram K Ellis W Keiser J. Antischistosomal Activities of Mefloquine-Related Arylmethanols. Antimicrob Agents Chemother (2012) 56:3207–15. 10.1128/AAC.06177-11 PMC3370792 22470113

[118] Ingram K Schiaffo CE Sittiwong W Benner E Dussault PH Keiser J. In Vitro and In Vivo Activity of 3-alkoxy-1,2-dioxolanes Against Schistosoma Mansoni. J Antimicrob Chemother (2012) 67:1979–86. 10.1093/jac/dks141 PMC3394440 22553141

[119] Al-Ali H. The Evolution of Drug Discovery: From Phenotypes to Targets, and Back. Medchemcomm (2016) 7:788–98. 10.1039/c6md00129g

[120] Heilker R Lessel U Bischoff D. The Power of Combining Phenotypic and Target-Focused Drug Discovery. Drug Discov Today (2019) 24:526–32. 10.1016/j.drudis.2018.10.009 30359770

[121] Lage OM Ramos MC Calisto R Almeida E Vasconcelos V Vicente F. Current Screening Methodologies in Drug Discovery for Selected Human Diseases. Mar Drugs (2018) 16:1–31. 10.3390/md16080279 PMC6117650 30110923

[122] Noël F do Monte FM. Validation of a Na+-shift Binding Assay for Estimation of the Intrinsic Efficacy of Ligands at the A2A Adenosine Receptor. J Pharmacol Toxicol Methods (2017) 84:51–6. 10.1016/j.vascn.2016.10.009 27810394

[123] Noël F Pompeu TET Moura BC. Functional Binding Assays for Estimation of the Intrinsic Efficacy of Ligands at the 5-HT1A Receptor: Application for Screening Drug Candidates. J Pharmacol Toxicol Methods (2014) 70:12–8. 10.1016/j.vascn.2014.03.002 24636913

[124] De Jong LAA Uges DRA Franke JP Bischoff R. Receptor-Ligand Binding Assays: Technologies and Applications. J Chromatogr B Anal Technol BioMed Life Sci (2005) 829:1–25. 10.1016/j.jchromb.2005.10.002 16253574

[125] Ashok Hajare A Sachin Salunkhe S Sachin MS Sonali Gorde S Sammer Nadaf J Sachi Pishawikar A. Review on : High-throughput Screening is an Approach to Drug Discovery. Am J Pharmtech Res (2014) 4:113–29.

[126] Rohman M Wingfield J. High-Throughput Screening Using Mass Spectrometry Within Drug Discovery. In: Methods in Molecular Biology. New York, NY, USA: Humana Press Inc (2016). p. 47–63. 10.1007/978-1-4939-3673-1_3 27316987

[127] Henderson MJ Holbert MA Simeonov A Kallal LA. High-Throughput Cellular Thermal Shift Assays in Research and Drug Discovery. SLAS Discov (2020) 25:137–47. 10.1177/2472555219877183 PMC10915787 31566060

[128] De Simone A Naldi M Bartolini M Davani L Andrisano V. Immobilized Enzyme Reactors: An Overview of Applications in Drug Discovery From 2008 to 2018. Chromatographia (2019) 82:425–41. 10.1007/s10337-018-3663-5

[129] Moraes MC Cardoso C Seidl C Moaddel R Cass Q. Targeting Anti-Cancer Active Compounds: Affinity-Based Chromatographic Assays. Curr Pharm Des (2016) 22:5976–87. 10.2174/1381612822666160614080506 PMC5154774 27306095

[130] Imaduwage KP Lakbub J Go EP Desaire H. Rapid LC-MS Based High-Throughput Screening Method, Affording No False Positives or False Negatives, Identifies a New Inhibitor for Carbonic Anhydrase. Sci Rep (2017) 7:1–10. 10.1038/s41598-017-08602-w 28871149 PMC5583356

[131] Wang L Zhao Y Zhang Y Zhang T Kool J Somsen GW. Online Screening of Acetylcholinesterase Inhibitors in Natural Products Using Monolith-Based Immobilized Capillary Enzyme Reactors Combined With Liquid Chromatography-Mass Spectrometry. J Chromatogr A (2018) 1563:135–43. 10.1016/j.chroma.2018.05.069 29866504

[132] Ferreira Lopes Vilela A Cardoso CL. An on-Flow Assay for Screening of β-Secretase Ligands by Immobilised Capillary Reactor-Mass Spectrometry. Anal Methods (2017) 9:2189–96. 10.1039/c7ay00284j

[133] Rodrigues MVN Rodrigues-Silva C Boaventura S Oliveira ASS Rath S Cass QB. On-Flow LC-MS/MS Method for Screening of Xanthine Oxidase Inhibitors. J Pharm BioMed Anal (2020) 181:113097. 10.1016/j.jpba.2020.113097 31931446

[134] Zhuo T Zhou S Zhang W Lambertucci C Volpini R. Synthesis and Ability of New Ligands for G Protein-Coupled Receptors 17 (GPR17). Med Sci Monit (2017) 23:953–9. 10.12659/MSM.902048 PMC5333714 28223679

[135] Kalinin DV Jana SK Pfafenrot M Chakrabarti A Melesina J Shaik TB. Structure-Based Design, Synthesis, and Biological Evaluation of Triazole-Based smHDAC8 Inhibitors. ChemMedChem (2020) 15:571–84. 10.1002/cmdc.201900583 PMC7187165 31816172

[136] Monaldi D Rotili D Lancelot J Marek M Wössner N Lucidi A. Structure-Reactivity Relationships on Substrates and Inhibitors of the Lysine Deacylase Sirtuin 2 From Schistosoma Mansoni (SmSirt2). J Med Chem (2019) 62:8733–59. 10.1021/acs.jmedchem.9b00638 31496251

[137] Li T Ziniel PD He P Kommer VP Crowther GJ He M. High-Throughput Screening Against Thioredoxin Glutathione Reductase Identifies Novel Inhibitors With Potential Therapeutic Value for Schistosomiasis. Infect Dis Poverty (2015) 4:40. 10.1186/s40249-015-0071-z 26341081 PMC4560900

[138] Lyu H Petukhov PA Banta PR Jadhav A Lea WA Cheng Q. Characterization of Lead Compounds Targeting the Selenoprotein Thioredoxin Glutathione Reductase for Treatment of Schistosomiasis. ACS Infect Dis (2020) 6:393–405. 10.1021/acsinfecdis.9b00354 31939288 PMC7072008

[139] Liu J Dyer D Wang J Wang S Du X Xu B. 3-oxoacyl-ACP Reductase From Schistosoma Japonicum: Integrated In Silico-In Vitro Strategy for Discovering Antischistosomal Lead Compounds. PloS One (2013) 8:e64984. 10.1371/journal.pone.0064984 23762275 PMC3676400

[140] Botros SS William S Sabra ANA El-Lakkany NM Seif el-Din SH García-Rubia A. Screening of a PDE-focused Library Identifies Imidazoles With In Vitro and In Vivo Antischistosomal Activity. Int J Parasitol Drugs Drug Resist (2019) 9:35–43. 10.1016/j.ijpddr.2019.01.001 30669086 PMC6350229

[141] Sebastián-Pérez V Schroeder S Munday JC Van Der Meer T Zaldívar-Díez J Siderius M. Discovery of Novel Schistosoma Mansoni PDE4A Inhibitors as Potential Agents Against Schistosomiasis. Future Med Chem (2019) 11:1703–20. 10.4155/fmc-2018-0592 31370708

[142] Jurberg AD Brindley PJ. Gene Function in Schistosomes: Recent Advances Towards a Cure. Front Genet (2015) 6:1–4. 10.3389/fgene.2015.00144 25926850 PMC4397921

[143] Huang W Gu M Cheng W Zhao QP Ming Z Dong H. Characteristics and Function of Cathepsin L3 From Schistosoma Japonicum. Parasitol Res (2020) 119:1619–28. 10.1007/s00436-020-06647-x 32185481

[144] Caffrey CR Placha L Barinka C Hradilek M Dostál J Sajid M. Homology Modeling and SAR Analysis of Schistosoma Japonicum Cathepsin D (SjCD) With Statin Inhibitors Identify a Unique Active Site Steric Barrier With Potential for the Design of Specific Inhibitors. Biol Chem (2005) 386:339–49. 10.1515/BC.2005.041 15899696

[145] Ekici ÖD Götz MG James KE Li ZZ Rukamp BJ Asgian JL. Aza-Peptide Michael Acceptors: A New Class of Inhibitors Specific for Caspases and Other Clan CD Cysteine Proteases. J Med Chem (2004) 47:1889–92. 10.1021/jm049938j 15055989

[146] Ovat A Muindi F Fagan C Brouner M Hansell E Dvořák J. Aza-Peptidyl Michael Acceptor and Epoxide Inhibitors—Potent and Selective Inhibitors of Schistosoma Mansoni and Ixodes Ricinus Legumains (Asparaginyl Endopeptidases). J Med Chem (2009) 52:7192–210. 10.1021/jm900849h 19848405

[147] Götz MG James KE Hansell E Dvořák J Seshaadri A Sojka D. Aza-Peptidyl Michael Acceptors. A New Class of Potent and Selective Inhibitors of Asparaginyl Endopeptidases (Legumains) From Evolutionarily Diverse Pathogens. J Med Chem (2008) 51:2816–32. 10.1021/jm701311r 18416543

[148] Fonseca NC da Cruz LF da Silva Villela F do Nascimento Pereira GA de Siqueira-Neto JL Kellar D. Synthesis of a Sugar-Based Thiosemicarbazone Series and Structure-Activity Relationship Versus the Parasite Cysteine Proteases Rhodesain, Cruzain, and Schistosoma Mansoni Cathepsin B1. Antimicrob Agents Chemother (2015) 59:2666–77. 10.1128/AAC.04601-14 PMC4394791 25712353

[149] Jílková A Horn M Fanfrlík J Küppers J Pachl P Řezáčová P. Azanitrile Inhibitors of the SmCB1 Protease Target are Lethal to Schistosoma Mansoni : Structural and Mechanistic Insights Into Chemotype Reactivity. ACS Infect Dis (2021) 7:189–201. 10.1021/acsinfecdis.0c00644 33301315 PMC7802074

[150] Jílková A Rubešová P Fanfrlík J Fajtová P Řezáčová P Brynda J. Druggable Hot Spots in the Schistosomiasis Cathepsin B1 Target Identified by Functional and Binding Mode Analysis of Potent Vinyl Sulfone Inhibitors. ACS Infect Dis (2020). 10.1021/acsinfecdis.0c00501 PMC8154419 33175511

[151] Horn M Jílková A Vondrášek J Marešová L Caffrey CR Mareš M. Mapping the Pro-Peptide of the Schistosoma Mansoni Cathepsin B1 Drug Target: Modulation of Inhibition by Heparin and Design of Mimetic Inhibitors. ACS Chem Biol (2011) 6:609–17. 10.1021/cb100411v 21375333

[152] Jílková A Řezáčová P Lepšík M Horn M Váchová J Fanfrlík J. Structural Basis for Inhibition of Cathepsin B Drug Target From the Human Blood Fluke, Schistosoma Mansoni. J Biol Chem (2011) 286:35770–81. 10.1074/jbc.M111.271304 PMC3195637 21832058

[153] Araujo-Montoya BO Senger MR Gomes BF Harris G Owens RJ Silva-Jr FP. Schistosoma Mansoni Cathepsin D1: Biochemical and Biophysical Characterization of the Recombinant Enzyme Expressed in HEK293T Cells. Protein Expr Purif (2020) 167:105532. 10.1016/j.pep.2019.105532 31711796

[154] Brady CP Dowd AJ Brindley PJ Ryan T Day SR Dalton JP. Recombinant Expression and Localization ofSchistosoma Mansoni Cathepsin L1 Support its Role in the Degradation of Host Hemoglobin. Infect Immun (1999) 67:368–74. 10.1128/IAI.67.1.368-374.1999 PMC96319 9864238

[155] Dvořák J Mashiyama ST Sajid M Braschi S Delcroix M Schneider EL. SmCL3, a Gastrodermal Cysteine Protease of the Human Blood Fluke Schistosoma Mansoni. PloS Negl Trop Dis (2009) 3:e449. 10.1371/journal.pntd.0000449 19488406 PMC2685030

[156] Bezerra IC Jorge MSB Gondim APS De LLL Vasconcelos MGF. “Fui Lá No Posto E O Doutor Me Mandou Foi Pra Cá”: Processo De Medicamentalização E (Des)Caminhos Para O Cuidado Em Saúde Mental Na Atenção Primária. Interface - Comun Saúde Educ (2014) 18:61–74. 10.1590/1807-57622013.0650

[157] Berriman M Haas BJ LoVerde PT Wilson RA Dillon GP Cerqueira GC. The Genome of the Blood Fluke Schistosoma Mansoni. Nature (2009) 460:352–8. 10.1038/nature08160 PMC2756445 19606141

[158] Grevelding CG Langner S Dissous C. Kinases: Molecular Stage Directors for Schistosome Development and Differentiation. Trends Parasitol (2018) 34:246–60. 10.1016/j.pt.2017.12.001 29276074

[159] Fioravanti R Mautone N Rovere A Rotili D Mai A. Targeting Histone Acetylation/Deacetylation in Parasites: An Update (2017–2020). Curr Opin Chem Biol (2020) 57:65–74. 10.1016/j.cbpa.2020.05.008 32615359

[160] Perera TPS Jovcheva E Mevellec L Vialard J De Lange D Verhulst T. Discovery and Pharmacological Characterization of JNJ-42756493 (Erdafitinib), a Functionally Selective Small-Molecule FGFR Family Inhibitor. Mol Cancer Ther (2017) 16:1010–20. 10.1158/1535-7163.MCT-16-0589 28341788

[161] Morales ME Rinaldi G Gobert GN Kines KJ Tort JF Brindley PJ. RNA Interference of Schistosoma Mansoni Cathepsin D, the Apical Enzyme of the Hemoglobin Proteolysis Cascade. Mol Biochem Parasitol (2008) 157:160–8. 10.1016/j.molbiopara.2007.10.009 PMC4130333 18067980

[162] Jílková A Horn M Mareš M. Structural and Functional Characterization of Schistosoma Mansoni Cathepsin B1. In: 1 ed. Schistosoma Mansoni: Methods and Protocols. New York, NY, USA: Springer US (2020). p. 145–58. 10.1007/978-1-0716-0635-3_12 32452002

[163] Fanfrlík J Brahmkshatriya PS Řezáč J Jílková A Horn M Mareš M. Quantum Mechanics-Based Scoring Rationalizes the Irreversible Inactivation of Parasitic Schistosoma Mansoni Cysteine Peptidase by Vinyl Sulfone Inhibitors. J Phys Chem B (2013) 117:14973–82. 10.1021/jp409604n 24195769

[164] Buro C Oliveira KC Lu Z Leutner S Beckmann S Dissous C. Transcriptome Analyses of Inhibitor-Treated Schistosome Females Provide Evidence for Cooperating Src-kinase and TGFβ Receptor Pathways Controlling Mitosis and Eggshell Formation. PloS Pathog (2013) 9:e1003448. 10.1371/journal.ppat.1003448 23785292 PMC3681755

[165] Hahnel S Quack T Parker-Manuel SJ Lu Z Vanderstraete M Morel M. Gonad RNA-specific qRT-PCR Analyses Identify Genes With Potential Functions in Schistosome Reproduction Such as SmFz1 and SmFGFRs. Front Genet (2014) 5:170. 10.3389/fgene.2014.00170 24959172 PMC4050651

[166] You H Stephenson RJ Gobert GN McManus DP. Revisiting Glucose Uptake and Metabolism in Schistosomes: New Molecular Insights for Improved Schistosomiasis Therapies. Front Genet (2014) 5:176. 10.3389/fgene.2014.00176 24966871 PMC4052099

[167] Chan JD McCorvy JD Acharya S Johns ME Day TA Roth BL. A Miniaturized Screen of a Schistosoma Mansoni Serotonergic G Protein-Coupled Receptor Identifies Novel Classes of Parasite-Selective Inhibitors. PloS Pathog (2016) 12:1–26. 10.1371/journal.ppat.1005651 PMC4871480 27187180

[168] Shuker SB Hajduk PJ Meadows RP Fesik SW. Discovering High-Affinity Ligands for Proteins: SAR by NMR. Science (1996) 274:1531–4. 10.1126/science.274.5292.1531 8929414

[169] Rees DC Congreve M Murray CW Carr R. Fragment-Based Lead Discovery. Nat Rev Drug Discov (2004) 3:660–72. 10.1038/nrd1467 15286733

[170] Schulz MN Hubbard RE. Recent Progress in Fragment-Based Lead Discovery. Curr Opin Pharmacol (2009) 9:615–21. 10.1016/j.coph.2009.04.009 19477685

[171] Erlanson DA Fesik SW Hubbard RE Jahnke W Jhoti H. Twenty Years on: The Impact of Fragments on Drug Discovery. Nat Rev Drug Discov (2016) 15:605–19. 10.1038/nrd.2016.109 27417849

[172] Erlanson DA De Esch IJP Jahnke W Johnson CN Mortenson PN. Fragment-to-Lead Medicinal Chemistry Publications in 2018. J Med Chem (2020) 63:4430–44. 10.1021/acs.jmedchem.9b01581 31913033

[173] Osborne J Panova S Rapti M Urushima T Jhoti H. Fragments: Where are We Now? Biochem Soc Trans (2020) 48:271–80. 10.1042/BST20190694 31985743

[174] Bollag G Tsai J Zhang J Zhang C Ibrahim P Nolop K. Vemurafenib: The First Drug Approved for BRAF-mutant Cancer. Nat Rev Drug Discov (2012) 11:873–86. 10.1038/nrd3847 23060265

[175] Souers AJ Leverson JD Boghaert ER Ackler SL Catron ND Chen J. ABT-199, a Potent and Selective BCL-2 Inhibitor, Achieves Antitumor Activity While Sparing Platelets. Nat Med (2013) 19:202–8. 10.1038/nm.3048 23291630

[176] Tap WD Wainberg ZA Anthony SP Ibrahim PN Zhang C Healey JH. Structure-Guided Blockade of CSF1R Kinase in Tenosynovial Giant-Cell Tumor. N Engl J Med (2015) 373:428–37. 10.1056/nejmoa1411366 26222558

[177] Bancet A Raingeval C Lomberget T Le Borgne M Guichou J-F Krimm I. Fragment Linking Strategies for Structure-Based Drug Design. J Med Chem (2020) 63:11420–35. 10.1021/acs.jmedchem.0c00242 32539387

[178] Congreve M Carr R Murray C Jhoti H. A ‘Rule of Three’ for Fragment-Based Lead Discovery? Drug Discov Today (2003) 8:876–7. 10.1016/S1359-6446(03)02831-9 14554012

[179] Giordanetto F Jin C Willmore L Feher M Shaw DE. Fragment Hits: What do They Look Like and How do They Bind? J Med Chem (2019) 62:3381–94. 10.1021/acs.jmedchem.8b01855 PMC6466478 30875465

[180] Hall RJ Mortenson PN Murray CW. Efficient Exploration of Chemical Space by Fragment-Based Screening. Prog Biophys Mol Biol (2014) 116:82–91. 10.1016/j.pbiomolbio.2014.09.007 25268064

[181] Hann MM Leach AR Harper G. Molecular Complexity and Its Impact on the Probability of Finding Leads for Drug Discovery. J Chem Inf Comput Sci (2001) 41:856–64. 10.1021/ci000403i 11410068

[182] Ferenczy GG Keserű GM. On the Enthalpic Preference of Fragment Binding. Medchemcomm (2016) 7:332–7. 10.1039/C5MD00542F

[183] Hopkins AL Keserü GM Leeson PD Rees DC Reynolds CH. The Role of Ligand Efficiency Metrics in Drug Discovery. Nat Rev Drug Discov (2014) 13:105–21. 10.1038/nrd4163 24481311

[184] Davis BJ Roughley SD. Fragment-Based Lead Discovery. 1st ed. Annual Reports in Medicinal Chemistry. Cambridge, MA, USA: Elsevier Inc (2017). 10.1016/bs.armc.2017.07.002

[185] Bulfer SL Jean-Francois FL Arkin MR. Making FBDD Work in Academia. In: Erlanson DA Jahnke W, editors. Fragment-Based Drug Discovery: Lessons and Outlook. Weinheim, Germany: Wiley-VCH Verlag GmbH & Co. KGaA (2016). p. 223–46. 10.1002/9783527683604.ch10

[186] Mello JDFRE Gomes RA Vital-Fujii DG Ferreira GM Trossini GHG. Fragment-Based Drug Discovery as Alternative Strategy to the Drug Development for Neglected Diseases. Chem Biol Drug Des (2017) 90:1067–78. 10.1111/cbdd.13030 28547936

[187] Keeley A Petri L Ábrányi-Balogh P Keserű GM. Covalent Fragment Libraries in Drug Discovery. Drug Discov Today (2020) 25:983–96. 10.1016/j.drudis.2020.03.016 32298798

[188] Keseru GM Erlanson DA Ferenczy GG Hann MM Murray CW Pickett SD. Design Principles for Fragment Libraries: Maximizing the Value of Learnings From Pharma Fragment-Based Drug Discovery (FBDD) Programs for Use in Academia. J Med Chem (2016) 59:8189–206. 10.1021/acs.jmedchem.6b00197 27124799

[189] Troelsen NS Clausen MH. Library Design Strategies To Accelerate Fragment-Based Drug Discovery. Chem - A Eur J (2020) 26:11391–403. 10.1002/chem.202000584 32339336

[190] Fragment Screening - Xchem. Available at: https://www.diamond.ac.uk/Instruments/Mx/Fragment-Screening.html (Accessed December 11, 2020).

[191] Cox OB Krojer T Collins P Monteiro O Talon R Bradley A. A Poised Fragment Library Enables Rapid Synthetic Expansion Yielding the First Reported Inhibitors of PHIP(2), an Atypical Bromodomain. Chem Sci (2016) 7:2322–30. 10.1039/c5sc03115j PMC5977933 29910922

[192] Roughley SD Jordan AM. The Medicinal Chemist ‘ s Toolbox : An Analysis of Reactions Used in the Pursuit of Novel Drug Candidates. J Med Chem (2011) 54:3451–79. 10.1021/jm200187y 21504168

[193] Douangamath A Fearon D Gehrtz P Krojer T Lukacik P Owen CD. Crystallographic and Electrophilic Fragment Screening of the SARS-CoV-2 Main Protease. Nat Commun (2020) 11:1–11. 10.1038/s41467-020-18709-w 33028810 PMC7542442

[194] Erlanson DA. Many Small Steps Towards a COVID-19 Drug. Nat Commun (2020) 11:1–4. 10.1038/s41467-020-18710-3 33028832 PMC7541474

[195] Coyle J Walser R. Applied Biophysical Methods in Fragment-Based Drug Discovery. SLAS Discov (2020) 25:471–90. 10.1177/2472555220916168 32345095

[196] Begley DW Moen SO Pierce PG Zartler ER. Saturation Transfer Difference NMR for Fragment Screening. In: . Current Protocols in Chemical Biology. Hoboken, NJ, USA: John Wiley & Sons, Inc (2013). p. 251–68. 10.1002/9780470559277.ch130118 24391096

[197] Becker W Bhattiprolu KC Gubensäk N Zangger K. Investigating Protein-Ligand Interactions by Solution Nuclear Magnetic Resonance Spectroscopy. ChemPhysChem (2018) 19:895–906. 10.1002/cphc.201701253 29314603 PMC5915746

[198] Li Q. Application of Fragment-Based Drug Discovery to Versatile Targets. Front Mol Biosci (2020) 7:180. 10.3389/fmolb.2020.00180 32850968 PMC7419598

[199] Arroyo X Goldflam M Feliz M Belda I Giralt E. Computer-Aided Design of Fragment Mixtures for NMR-Based Screening. PloS One (2013) 8:e58571. 10.1371/journal.pone.0058571 23516512 PMC3596306

[200] Navratilova I Hopkins AL. Fragment Screening by Surface Plasmon Resonance. ACS Med Chem Lett (2010) 1:44–8. 10.1021/ml900002k PMC4007845 24900174

[201] Giannetti AM. From Experimental Design to Validated Hits a Comprehensive Walk-Through of Fragment Lead Identification Using Surface Plasmon Resonance. In: 1st ed. Methods in Enzymology. Cambridge, MA, USA: Elsevier Inc (2011). 10.1016/B978-0-12-381274-2.00008-X 21371592

[202] Giannetti AM Koch BD Browner MF. Surface Plasmon Resonance Based Assay for the Detection and Characterization of Promiscuous Inhibitors. J Med Chem (2008) 51:574–80. 10.1021/jm700952v 18181566

[203] Niesen FH Berglund H Vedadi M. The Use of Differential Scanning Fluorimetry to Detect Ligand Interactions That Promote Protein Stability. Nat Protoc (2007) 2:2212–21. 10.1038/nprot.2007.321 17853878

[204] Kranz JK Schalk-Hihi C. Protein Thermal Shifts to Identify Low Molecular Weight Fragments. In: 1st ed. Methods in Enzymology. Cambridge, MA, USA: Elsevier Inc (2011). 10.1016/B978-0-12-381274-2.00011-X 21371595

[205] Kirsch P Hartman AM Hirsch AKH Empting M. Concepts and Core Principles of Fragment-Based Drug Design. Molecules (2019) 24:4309. 10.3390/molecules24234309 PMC6930586 31779114

[206] Collins PM Douangamath A Talon R Dias A Brandao-Neto J Krojer T. Achieving a Good Crystal System for Crystallographic X-Ray Fragment Screening. In: 1st ed. Methods in Enzymology. Cambridge, MA, USA: Elsevier Inc (2018). 10.1016/bs.mie.2018.09.027 30390801

[207] Hassell AM An G Bledsoe RK Bynum JM Carter HL Deng SJJ. Crystallization of Protein-Ligand Complexes. Acta Crystallogr Sect D Biol Crystallogr (2006) 63:72–9. 10.1107/S0907444906047020 PMC2483499 17164529

[208] Danley DE. Crystallization to Obtain Protein-Ligand Complexes for Structure-Aided Drug Design. Acta Crystallogr Sect D Biol Crystallogr (2006) 62:569–75. 10.1107/S0907444906012601 16699182

[209] Hoffer L Muller C Roche P Morelli X. Chemistry-Driven Hit-to-lead Optimization Guided by Structure-based Approaches. Mol Inform (2018) 37:1800059. 10.1002/minf.201800059 30051601

[210] Murray CW Rees DC. The Rise of Fragment-Based Drug Discovery. Nat Chem (2009) 1:187–92. 10.1038/nchem.217 21378847

[211] Chilingaryan Z Yin Z Oakley AJ. Fragment-Based Screening by Protein Crystallography: Successes and Pitfalls. Int J Mol Sci (2012) 13:12857–79. 10.3390/ijms131012857 PMC3497300 23202926

[212] Murray CW Rees DC. Opportunity Knocks: Organic Chemistry for Fragment-Based Drug Discovery (FBDD). Angew Chem Int Ed (2016) 55:488–92. 10.1002/anie.201506783 26526786

[213] Valenti D Hristeva S Tzalis D Ottmann C. Clinical Candidates Modulating Protein-Protein Interactions: The Fragment-Based Experience. Eur J Med Chem (2019) 167:76–95. 10.1016/j.ejmech.2019.01.084 30769242

[214] Hopkins AL Groom CR Alex A. Ligand Efficiency: A Useful Metric for Lead Selection. Drug Discov Today (2004) 9:430–1. 10.1016/S1359-6446(04)03069-7 15109945

[215] Kumar S Waldo JP Jaipuri FA Marcinowicz A Van Allen C Adams J. Discovery of Clinical Candidate (1 R,4 R)-4-((R)-2-((S)-6-Fluoro-5 H-imidazo[5,1-A[isoindol-5-yl)-1-hydroxyethyl)cyclohexan-1-ol (Navoximod), a Potent and Selective Inhibitor of Indoleamine 2,3-Dioxygenase 1. J Med Chem (2019) 62:6705–33. 10.1021/acs.jmedchem.9b00662 31264862

[216] Hudson SA McLean KJ Surade S Yang YQ Leys D Ciulli A. Application of Fragment Screening and Merging to the Discovery of Inhibitors of the Mycobacterium Tuberculosis Cytochrome P450 CYP121. Angew Chem Int Ed (2012) 51:9311–6. 10.1002/anie.201202544 22890978

[217] Hung AW Silvestre HL Wen S Ciulli A Blundell TL Abell C. Application of Fragment Growing and Fragment Linking to the Discovery of Inhibitors of Mycobacterium Tuberculosis Pantothenate Synthetase. Angew Chem Int Ed (2009) 48:8452–6. 10.1002/anie.200903821 19780086

[218] Lamoree B Hubbard RE. Current Perspectives in Fragment-Based Lead Discovery (FBLD). Essays Biochem (2017) 61:453–64. 10.1042/EBC20170028 PMC5869234 29118093

[219] Kidd SL Osberger TJ Mateu N Sore HF Spring DR. Recent Applications of Diversity-Oriented Synthesis Toward Novel, 3-Dimensional Fragment Collections. Front Chem (2018) 6:460. 10.3389/fchem.2018.00460 30386766 PMC6198038

[220] Drwal MN Bret G Perez C Jacquemard C Desaphy J Kellenberger E. Structural Insights on Fragment Binding Mode Conservation. J Med Chem (2018) 61:5963–73. 10.1021/acs.jmedchem.8b00256 29906118

[221] Scott DE Coyne AG Hudson SA Abell C. Fragment-Based Approaches in Drug Discovery and Chemical Biology. Biochemistry (2012) 51:4990–5003. 10.1021/bi3005126 22697260

[222] Singh M Tam B Akabayov B. NMR-Fragment Based Virtual Screening: A Brief Overview. Molecules (2018) 23:233. 10.3390/molecules23020233 PMC6017141 29370102

[223] Miyake Y Itoh Y Hatanaka A Suzuma Y Suzuki M Kodama H. Identification of Novel Lysine Demethylase 5-Selective Inhibitors by Inhibitor-Based Fragment Merging Strategy. Bioorg Med Chem (2019) 27:1119–29. 10.1016/j.bmc.2019.02.006 30745098

[224] Bian Y Xie X-Q. Computational Fragment-Based Drug Design: Current Trends, Strategies, and Applications. AAPS J (2018) 20:59. 10.1208/s12248-018-0216-7 29633051 PMC6618289

[225] Moreira-Filho JT Dantas RF Senger MR Silva AC Campos DMB Muratov E. Shortcuts to Schistosomiasis Drug Discovery: The State-of-the-Art. In: 1 ed. Annual Reports in Medicinal Chemistry. Cambridge, MA, USA: Elsevier (2019). p. 139–80. 10.1016/bs.armc.2019.06.004

[226] Nazaré M Matter H Will DW Wagner M Urmann M Czech J. Fragment Deconstruction of Small, Potent Factor Xa Inhibitors: Exploring the Superadditivity Energetics of Fragment Linking in Protein-Ligand Complexes. Angew Chem Int Ed (2012) 51:905–11. 10.1002/anie.201107091 22190348

[227] Mondal M Radeva N Fanlo-Virgós H Otto S Klebe G Hirsch AKH. Fragment Linking and Optimization of Inhibitors of the Aspartic Protease Endothiapepsin: Fragment-Based Drug Design Facilitated by Dynamic Combinatorial Chemistry. Angew Chem Int Ed (2016) 55:9422–6. 10.1002/anie.201603074 PMC5113778 27400756

[228] Kuntz AN Davioud-Charvet E Sayed AA Califf LL Dessolin J Arnér ESJ. Thioredoxin Glutathione Reductase From Schistosoma Mansoni: An Essential Parasite Enzyme and a Key Drug Target. PloS Med (2007) 4:e206. 10.1371/journal.pmed.0040206 17579510 PMC1892040

[229] Prast-Nielsen S Huang HH Williams DL. Thioredoxin Glutathione Reductase: Its Role in Redox Biology and Potential as a Target for Drugs Against Neglected Diseases. Biochim Biophys Acta - Gen Subj (2011) 1810:1262–71. 10.1016/j.bbagen.2011.06.024 PMC3210934 21782895

[230] Song L Li J Xie S Qian C Wang J Zhang W. Thioredoxin Glutathione Reductase as a Novel Drug Target: Evidence From Schistosoma Japonicum. PloS One (2012) 7:e31456. 10.1371/journal.pone.0031456 22384025 PMC3285170

[231] Simeonov A Jadhav A Sayed AA Wang Y Nelson ME Thomas CJ. Quantitative High-Throughput Screen Identifies Inhibitors of the Schistosoma Mansoni Redox Cascade. PloS Negl Trop Dis (2008) 2:e127. 10.1371/journal.pntd.0000127 18235848 PMC2217675

[232] Lea WA Jadhav A Rai G Sayed AA Cass CL Inglese J. A 1,536-Well-Based Kinetic HTS Assay for Inhibitors of Schistosoma Mansoni Thioredoxin Glutathione Reductase. Assay Drug Dev Technol (2008) 6:551–5. 10.1089/adt.2008.149 PMC2669305 18665782

[233] Silvestri I Lyu H Fata F Boumis G Miele AE Ardini M. Fragment-Based Discovery of a Regulatory Site in Thioredoxin Glutathione Reductase Acting as “Doorstop” for NADPH Entry. ACS Chem Biol (2018) 13:2190–202. 10.1021/acschembio.8b00349 PMC6905387 29800515

[234] Sheng C Zhang W. Fragment Informatics and Computational Fragment-Based Drug Design: An Overview and Update. Med Res Rev (2013) 33:554–98. 10.1002/med.21255 22430881

[235] Grove LE Vajda S Kozakov D. Computational Methods to Support Fragment-Based Drug Discovery. In: Erlanson DA Jahnke W, editors. Fragment-Based Drug Discovery: Lessons and Outlook. Weinheim, Germany: Wiley-VCH Verlag GmbH & Co. KGaA (2016). p. 197–222. 10.1002/9783527683604.ch09

[236] Gianti E Sartori L. Identification and Selection of “Privileged Fragments” Suitable for Primary Screening. J Chem Inf Model (2008) 48:2129–39. 10.1021/ci800219h 18991373

[237] Hoffer L Renaud JP Horvath D. In Silico Fragment-Based Drug Discovery: Setup and Validation of a Fragment-to-Lead Computational Protocol Using S4MPLE. J Chem Inf Model (2013) 53:836–51. 10.1021/ci4000163 23537132

[238] Barelier S Eidam O Fish I Hollander J Figaroa F Nachane R. Increasing Chemical Space Coverage by Combining Empirical and Computational Fragment Screens. ACS Chem Biol (2014) 9:1528–35. 10.1021/cb5001636 PMC4215856 24807704

[239] Kumar A Voet A Zhang KYJ. Fragment Based Drug Design: From Experimental to Computational Approaches. Curr Med Chem (2012) 19:5128–47. 10.2174/092986712803530467 22934764

[240] Rudling A Gustafsson R Almlöf I Homan E Scobie M Warpman Berglund U. Fragment-Based Discovery and Optimization of Enzyme Inhibitors by Docking of Commercial Chemical Space. J Med Chem (2017) 60:8160–9. 10.1021/acs.jmedchem.7b01006 28929756

[241] Lyu J Wang S Balius TE Singh I Levit A Moroz YS. Ultra-Large Library Docking for Discovering New Chemotypes. Nature (2019) 566:224–9. 10.1038/s41586-019-0917-9 PMC6383769 30728502

[242] Kleandrova VV Speck-Planche A. The QSAR Paradigm in Fragment-Based Drug Discovery: From the Virtual Generation of Target Inhibitors to Multi-Scale Modeling. Mini Rev Med Chem (2020) 20:1357–74. 10.2174/1389557520666200204123156 32013845

[243] de Souza Neto LR Moreira-Filho JT Neves BJ Maidana RLBR Guimarães ACR Furnham N. In Silico Strategies to Support Fragment-to-Lead Optimization in Drug Discovery. Front Chem (2020) 8:93. 10.3389/fchem.2020.00093 32133344 PMC7040036

[244] Erlanson DA Davis BJ Jahnke W. Fragment-Based Drug Discovery: Advancing Fragments in the Absence of Crystal Structures. Cell Chem Biol (2019) 26:9–15. 10.1016/j.chembiol.2018.10.001 30482678

[245] Bissaro M Sturlese M Moro S. The Rise of Molecular Simulations in Fragment-Based Drug Design (FBDD): An Overview. Drug Discov Today (2020) 25:1693–701. 10.1016/j.drudis.2020.06.023 PMC7314695 32592867

[246] Protasio AV Tsai IJ Babbage A Nichol S Hunt M Aslett MA. A Systematically Improved High Quality Genome and Transcriptome of the Human Blood Fluke Schistosoma Mansoni. PloS Negl Trop Dis (2012) 6:e1455. 10.1371/journal.pntd.0001455 22253936 PMC3254664

[247] Young ND Jex AR Li B Liu S Yang L Xiong Z. Whole-Genome Sequence of Schistosoma Haematobium. Nat Genet (2012) 44:221–5. 10.1038/ng.1065 22246508

[248] Zhou Y Zheng H Chen Y Zhang L Wang K Guo J. The Schistosoma Japonicum Genome Reveals Features of Host–Parasite Interplay. Nature (2009) 460:345–51. 10.1038/nature08140 PMC3747554 19606140

[249] Luo F Yin M Mo X Sun C Wu Q Zhu B. An Improved Genome Assembly of the Fluke Schistosoma Japonicum. PloS Negl Trop Dis (2019) 13:e0007612. 10.1371/journal.pntd.0007612 31390359 PMC6685614

[250] Stroehlein AJ Korhonen PK Chong TM Lim YL Chan KG Webster B. High-Quality Schistosoma Haematobium Genome Achieved by Single-Molecule and Long-Range Sequencing. Gigascience (2019) 8:1–12. 10.1093/gigascience/giz108 PMC6736295 31494670

[251] Mitchell AL Attwood TK Babbitt PC Blum M Bork P Bridge A. InterPro in 2019: Improving Coverage, Classification and Access to Protein Sequence Annotations. Nucleic Acids Res (2019) 47:D351–60. 10.1093/nar/gky1100 PMC6323941 30398656

[252] Lam SD Dawson NL Das S Sillitoe I Ashford P Lee D. Gene3D: Expanding the Utility of Domain Assignments. Nucleic Acids Res (2016) 44:D404–9. 10.1093/nar/gkv1231 PMC4702871 26578585

[253] Sillitoe I Lewis TE Cuff A Das S Ashford P Dawson NL. CATH: Comprehensive Structural and Functional Annotations for Genome Sequences. Nucleic Acids Res (2015) 43:D376–81. 10.1093/nar/gku947 PMC4384018 25348408

[254] Lu S Wang J Chitsaz F Derbyshire MK Geer RC Gonzales NR. CDD/SPARCLE: The Conserved Domain Database in 2020. Nucleic Acids Res (2020) 48:D265–8. 10.1093/nar/gkz991 PMC6943070 31777944

[255] Thomas PD Campbell MJ Kejariwal A Mi H Karlak B Daverman R. PANTHER: A Library of Protein Families and Subfamilies Indexed by Function. Genome Res (2003) 13:2129–41. 10.1101/gr.772403 PMC403709 12952881

[256] Finn RD Coggill P Eberhardt RY Eddy SR Mistry J Mitchell AL. The Pfam Protein Families Database: Towards a More Sustainable Future. Nucleic Acids Res (2015) 44:D279–85. 10.1093/nar/gkv1344 PMC4702930 26673716

[257] Mistry J Chuguransky S Williams L Qureshi M Salazar GA Sonnhammer ELL. Pfam: The Protein Families Database in 2021. Nucleic Acids Res (2021) 49:D412–9. 10.1093/nar/gkaa913 PMC7779014 33125078

[258] Sigrist CJA Cerutti L Hulo N Gattiker A Falquet L Pagni M. Prosite: A Documented Database Using Patterns and Profiles as Motif Descriptors. Brief Bioinform (2002) 3:265–74. 10.1093/bib/3.3.265 12230035

[259] Attwood TK Coletta A Muirhead G Pavlopoulou A Philippou PB Popov I. The PRINTS Database: A Fine-Grained Protein Sequence Annotation and Analysis Resource-its Status in 2012. Database (2012) 2012:1–9. 10.1093/database/bas019 PMC3326521 22508994

[260] Letunic I Khedkar S Bork P. SMART: Recent Updates, New Developments and Status in 2020. Nucleic Acids Res (2021) 49:D458–60. 10.1093/nar/gkaa937 PMC7778883 33104802

[261] Akiva E Brown S Almonacid DE Barber AE Custer AF Hicks MA. The Structure-Function Linkage Database. Nucleic Acids Res (2014) 42:521–30. 10.1093/nar/gkt1130 PMC3965090 24271399

[262] Pandurangan AP Stahlhacke J Oates ME Smithers B Gough J. The SUPERFAMILY 2.0 Database: A Significant Proteome Update and a New Webserver. Nucleic Acids Res (2019) 47:D490–4. 10.1093/nar/gky1130 PMC6324026 30445555

[263] Gough J Karplus K Hughey R Chothia C. Assignment of Homology to Genome Sequences Using a Library of Hidden Markov Models That Represent All Proteins of Known Structure. J Mol Biol (2001) 313:903–19. 10.1006/jmbi.2001.5080 11697912

[264] Haft DH Loftus BJ Richardson DL Yang F Eisen JA Paulsen IT. TIGRFAMs: A Protein Family Resource for the Functional Identification of Proteins. Nucleic Acids Res (2001) 29:41–3. 10.1093/nar/29.1.41 PMC29844 11125044

[265] Du J Li M Yuan Z Guo M Song J Xie X. A Decision Analysis Model for KEGG Pathway Analysis. BMC Bioinformatics (2016) 17:407. 10.1186/s12859-016-1285-1 27716040 PMC5053338

[266] Ashburner M Ball CA Blake JA Botstein D Butler H Cherry JM. Gene Ontology : Tool for the Unification of Biology. Nat Genet (2011) 25:25–9. 10.1038/75556 PMC3037419 10802651

[267] Furnham N de Beer TAP Thornton JM. Current Challenges in Genome Annotation Through Structural Biology and Bioinformatics. Curr Opin Struct Biol (2012) 22:594–601. 10.1016/j.sbi.2012.07.005 22884875

[268] Harris TW Arnaboldi V Cain S Chan J Chen WJ Cho J. WormBase: A Modern Model Organism Information Resource. Nucleic Acids Res (2019) 48:D762–7. 10.1093/nar/gkz920 PMC7145598 31642470

[269] Xia T Giri BR Liu J Du P Li X Li X. RNA Sequencing Analysis of Altered Expression of Long Noncoding RNAs Associated With Schistosoma Japonicum Infection in the Murine Liver and Spleen. Parasit Vectors (2020) 13:1–15. 10.1186/s13071-020-04457-9 33261628 PMC7705434

[270] Vasconcelos EJR Dasilva LF Pires DS Lavezzo GM Pereira ASA Amaral MS. The Schistosoma Mansoni Genome Encodes Thousands of Long non-Coding RNAs Predicted to be Functional at Different Parasite Life-Cycle Stages. Sci Rep (2017) 7:1–17. 10.1038/s41598-017-10853-6 28874839 PMC5585378

[271] Amaral MS Maciel LF Silveira GO Olberg GGO Leite JVP Imamura LK. Long non-Coding RNA Levels can be Modulated by 5-Azacytidine in Schistosoma Mansoni. Sci Rep (2020) 10:1–17. 10.1038/s41598-020-78669-5 33299037 PMC7725772

[272] Wendt G Zhao L Chen R Liu C O’Donoghue AJ Caffrey CR. A Single-Cell RNAseq Atlas of the Pathogenic Stage of Schistosoma Mansoni Identifies a Key Regulator of Blood Feeding. bioRxiv (2020) 1649:1644–9. 10.1101/2020.02.03.932004 PMC7875187 32973030

[273] Maciel LF Verjovski-Almeida S. Step-by-Step Bioinformatics Analysis of Schistosoma Mansoni Long non-Coding RNA Sequences. In: Timson DJ, editor. Methods in Molecular Biology. New York: Humana (2020). p. 109–33. 10.1007/978-1-0716-0635-3_10 32452000

[274] R Development Core Team. R: A Language and Environment for Statistical Computing. (2020).

[275] Kumar S Filipski AJ Battistuzzi FU Pond SLK Tamura K. Statistics and Truth in Phylogenomics. Mol Biol Evol (2012) 29:457–72. 10.1093/molbev/msr202 PMC3258035 21873298

[276] Koonin EV. Orthologs, Paralogs, and Evolutionary Genomics. Annu Rev Genet (2005) 39:309–38. 10.1146/annurev.genet.39.073003.114725 16285863

[277] Engelhardt BE Jordan MI Muratore KE Brenner SE. Protein Molecular Function Prediction by Bayesian Phylogenomics. PloS Comput Biol (2005) 1:e45. 10.1371/journal.pcbi.0010045 16217548 PMC1246806

[278] Brown D Sjo K. Functional Classification Using Phylogenomic Inference. PloS Comput Biol (2006) 2:e77. 10.1371/journal.pcbi.0020077 16846248 PMC1484587

[279] Guindon S Dufayard JF Lefort V Anisimova M Hordijk W Gascuel O. New Algorithms and Methods to Estimate Maximum-Likelihood Phylogenies: Assessing the Performance of PhyML 3.0. Syst Biol (2010) 59:307–21. 10.1093/sysbio/syq010 20525638

[280] Stamatakis A. RaxML Version 8: A Tool for Phylogenetic Analysis and Post-Analysis of Large Phylogenies. Bioinformatics (2014) 30:1312–3. 10.1093/bioinformatics/btu033 PMC3998144 24451623

[281] Price MN Dehal PS Arkin AP. FastTree 2 – Approximately Maximum-Likelihood Trees for Large Alignments. PloS One (2010) 5:e9490. 10.1371/journal.pone.0009490 20224823 PMC2835736

[282] Kind KK Sgaramella-zonta LA. Phylogenetic Analysis : Concepts and Methods. Am J Hum Genet (1971) 23:235–52. PMC1706731 5089842

[283] Zhou X Shen X-X Hittinger CT Rokas A. Evaluating Fast Maximum Likelihood-Based Phylogenetic Programs Using Empirical Phylogenomic Data Sets. Mol Biol Evol (2018) 35:486–503. 10.1093/molbev/msx302 29177474 PMC5850867

[284] Sebastian Strimmer K. Maximum Likelihood Methods in Molecular Phylogenetics. (1997).

[285] Penny D Hendy MD Steel MA. Progress With Methods for Constructing Evolutionary Trees. Trends Ecol Evol (1992) 7:73–9. 10.1016/0169-5347(92)90244-6 21235960

[286] Kumar S Stecher G Tamura K. MEGA7: Molecular Evolutionary Genetics Analysis Version 7.0 for Bigger Datasets. Mol Biol Evol (2016) 33:1870–4. 10.1093/molbev/msw054 PMC8210823 27004904

[287] Doolittle WF. Phylogenetic Classification and the Universal Tree. Science (1999) 284:2124–8. 10.1126/science.284.5423.2124 10381871

[288] Mullard A. 2018 FDA Drug Approvals. Nat Rev Drug Discov (2019) 18:85–9. 10.1038/d41573-019-00014-x 30710142

[289] Smietana K Siatkowski M Møller M. Trends in Clinical Success Rates. Nat Rev Drug Discov (2016) 15:379–80. 10.1038/nrd.2016.85 27199245

[290] Hopfinger A. Computer-Assisted Drug Design. J Med Chem (1985) 28:1133–9. 10.1021/jm00147a001 2993608

[291] Baig MH Ahmad K Roy S Ashraf JM Adil M Siddiqui MH. Computer Aided Drug Design: Success and Limitations. Curr Pharm Des (2016) 22:572–81. 10.2174/1381612822666151125000550 26601966

[292] Gasteiger J. Introduction. In: Gasteiger J Engel T, editors. Chemoinformatics. Weinheim, FRG: Wiley-VCH Verlag GmbH & Co. KGaA (2003). p. 1–13. 10.1002/3527601643.ch1

[293] Brown FK. Chemoinformatics: What is it and How Does it Impact Drug Discovery. In: 1 ed . Annual Reports in Medicinal Chemistry. Cambridge, MA, USA: Elsevier (1998). p. 375–84. 10.1016/S0065-7743(08)61100-8

[294] Engel T. Basic Overview of Chemoinformatics. J Chem Inf Model (2006) 46:2267–77. 10.1021/ci600234z 17125169

[295] Yang X Wang Y Byrne R Schneider G Yang S. Concepts of Artificial Intelligence for Computer-Assisted Drug Discovery. Chem Rev (2019) 119:10520–94. 10.1021/acs.chemrev.8b00728 31294972

[296] Schneider P Walters WP Plowright AT Sieroka N Listgarten J Goodnow RA. Rethinking Drug Design in the Artificial Intelligence Era. Nat Rev Drug Discov (2020) 19:353–64. 10.1038/s41573-019-0050-3 31801986

[297] Mater AC Coote ML. Deep Learning in Chemistry. J Chem Inf Model (2019) 59:2545–59. 10.1021/acs.jcim.9b00266 31194543

[298] Ekins S Puhl AC Zorn KM Lane TR Russo DP Klein JJ. Exploiting Machine Learning for End-to-End Drug Discovery and Development. Nat Mater (2019) 18:435–41. 10.1038/s41563-019-0338-z PMC6594828 31000803

[299] Caffrey CR El-Sakkary N Mäder P Krieg R Becker K Schlitzer M. Drug Discovery and Development for Schistosomiasis. In: 1 ed. Neglected Tropical Diseases: Drug Discovery and Development. Weinheim, Germany: Wiley‐VCH Verlag GmbH & Co. KGaA (2019). p. 187–225. 10.1002/9783527808656.ch8

[300] Zhu H. Big Data and Artificial Intelligence Modeling for Drug Discovery. Annu Rev Pharmacol Toxicol (2020) 60:573–89. 10.1146/annurev-pharmtox-010919-023324 PMC7010403 31518513

[301] Wang Y Bolton E Dracheva S Karapetyan K Shoemaker BA Suzek TO. An Overview of the PubChem BioAssay Resource. Nucleic Acids Res (2010) 38:D255–66. 10.1093/nar/gkp965 PMC2808922 19933261

[302] Wang Y Xiao J Suzek TO Zhang J Wang J Zhou Z. Pubchem’s BioAssay Database. Nucleic Acids Res (2012) 40:D400–12. 10.1093/nar/gkr1132 PMC3245056 22140110

[303] Kim S Chen J Cheng T Gindulyte A He J He S. PubChem in 2021: New Data Content and Improved Web Interfaces. Nucleic Acids Res (2021) 49:D1388–95. 10.1093/nar/gkaa971 PMC7778930 33151290

[304] Gaulton A Bellis LJ Bento AP Chambers J Davies M Hersey A. ChEMBL: A Large-Scale Bioactivity Database for Drug Discovery. Nucleic Acids Res (2012) 40:D1100–7. 10.1093/nar/gkr777 PMC3245175 21948594

[305] Duffy BC Zhu L Decornez H Kitchen DB. Early Phase Drug Discovery: Cheminformatics and Computational Techniques in Identifying Lead Series. Bioorg Med Chem (2012) 20:5324–42. 10.1016/j.bmc.2012.04.062 22938785

[306] Neves BJ Muratov E Machado RB Andrade CH Cravo PVL. Modern Approaches to Accelerate Discovery of New Antischistosomal Drugs. Expert Opin Drug Discov (2016) 11:557–67. 10.1080/17460441.2016.1178230 PMC6534417 27073973

[307] Martinez-Mayorga K Madariaga-Mazon A Medina-Franco JL Maggiora G. The Impact of Chemoinformatics on Drug Discovery in the Pharmaceutical Industry. Expert Opin Drug Discov (2020) 15:293–306. 10.1080/17460441.2020.1696307 31965870

[308] Chen H Kogej T Engkvist O. Cheminformatics in Drug Discovery, an Industrial Perspective. Mol Inform (2018) 37:1800041. 10.1002/minf.201800041 29774657

[309] Gasteiger J. Chemoinformatics: Achievements and Challenges, a Personal View. Molecules (2016) 21:151. 10.3390/molecules21020151 26828468 PMC6273366

[310] Humbeck L Koch O. What Can We Learn From Bioactivity Data? Chemoinformatics Tools and Applications in Chemical Biology Research. ACS Chem Biol (2017) 12:23–35. 10.1021/acschembio.6b00706 27779378

[311] Bajorath J. Foundations of Data-Driven Medicinal Chemistry. Futur Sci OA (2018) 4:FSO320. 10.4155/fsoa-2018-0057 PMC6153455 30271612

[312] Mak KK Pichika MR. Artificial Intelligence in Drug Development: Present Status and Future Prospects. Drug Discov Today (2019) 24:773–80. 10.1016/j.drudis.2018.11.014 30472429

[313] Schneider G. Automating Drug Discovery. Nat Rev Drug Discov (2017) 17:97–113. 10.1038/nrd.2017.232 29242609

[314] Lo Y-C Rensi SE Torng W Altman RB. Machine Learning in Chemoinformatics and Drug Discovery. Drug Discov Today (2018) 23:1538–46. 10.1016/j.drudis.2018.05.010 PMC6078794 29750902

[315] Schneider G. Mind and Machine in Drug Design. Nat Mach Intell (2019) 1:128–30. 10.1038/s42256-019-0030-7

[316] Griffen EJ Dossetter AG Leach AG. Chemists: AI is Here; Unite To Get the Benefits. J Med Chem (2020) 63:8695–704. 10.1021/acs.jmedchem.0c00163 32459965

[317] Vamathevan J Clark D Czodrowski P Dunham I Ferran E Lee G. Applications of Machine Learning in Drug Discovery and Development. Nat Rev Drug Discov (2019) 18:463–77. 10.1038/s41573-019-0024-5 PMC6552674 30976107

[318] Neves BJ Braga RC Melo-Filho CC Moreira-Filho JT Muratov EN Andrade CH. QSAR-Based Virtual Screening: Advances and Applications in Drug Discovery. Front Pharmacol (2018) 9:1275. 10.3389/fphar.2018.01275 30524275 PMC6262347

[319] Zhavoronkov A Vanhaelen Q Oprea TI. Will Artificial Intelligence for Drug Discovery Impact Clinical Pharmacology? Clin Pharmacol Ther (2020) 107:780–5. 10.1002/cpt.1795 PMC7158211 31957003

[320] Svetnik V Liaw A Tong C Culberson JC Sheridan RP Feuston BP. Random Forest: A Classification and Regression Tool for Compound Classification and QSAR Modeling. J Chem Inf Comput Sci (2003) 43:1947–58. 10.1021/ci034160g 14632445

[321] Breiman LEO. Random Forests. Mach Learn (2001) 45:5–32. 10.1023/A:1010933404324

[322] Cova TFGG Pais AACC. Deep Learning for Deep Chemistry: Optimizing the Prediction of Chemical Patterns. Front Chem (2019) 7:809. 10.3389/fchem.2019.00809 32039134 PMC6988795

[323] Tropsha A. Best Practices for QSAR Model Development, Validation, and Exploitation. Mol Inform (2010) 29:476–88. 10.1002/minf.201000061 27463326

[324] Fourches D Muratov E Tropsha A. Trust, But Verify: on the Importance of Chemical Structure Curation in Cheminformatics and QSAR Modeling Research. J Chem Inf Model (2010) 50:1189–204. 10.1021/ci100176x PMC2989419 20572635

[325] Fourches D Muratov E Tropsha A. Curation of Chemogenomics Data. Nat Chem Biol (2015) 11:535. 10.1038/nchembio.1881 26196763

[326] Fourches D Muratov EN Tropsha A. Trust, But Verify II: A Practical Guide to Chemogenomics Data Curation. J Chem Inf Model (2016) 56:1243–52. 10.1021/acs.jcim.6b00129 PMC5657146 27280890

[327] Danishuddin Khan AU. Descriptors and Their Selection Methods in QSAR Analysis: Paradigm for Drug Design. Drug Discov Today (2016) 21:1291–302. 10.1016/j.drudis.2016.06.013 27326911

[328] Todeschini R Consonni V. Molecular Descriptors for Chemoinformatics. Weinheim, Germany: WILEY-VCH (2009).

[329] Muratov EN Bajorath J Sheridan RP Tetko IV Filimonov D Poroikov V. QSAR Without Borders. Chem Soc Rev (2020) 49:3525–64. 10.1039/d0cs00098a PMC8008490 32356548

[330] Cherkasov A Muratov EN Fourches D Varnek A Baskin II Cronin M. QSAR Modeling: Where Have You Been? Where are You Going to? J Med Chem (2014) 57:4977–5010. 10.1021/jm4004285 24351051 PMC4074254

[331] Tropsha A Gramatica P Gombar VK. The Importance of Being Earnest: Validation is the Absolute Essential for Successful Application and Interpretation of QSPR Models. QuantStructActRelatCombSci (2003) 22:69–77. 10.1002/qsar.200390007

[332] Roy K Mitra I. On Various Metrics Used for Validation of Predictive QSAR Models With Applications in Virtual Screening and Focused Library Design. Comb Chem High Throughput Screen (2011) 14:450–74. 10.2174/138620711795767893 21521150

[333] OECD Principles for the Validation, for Regulatory Purposes, of (Quantitative) Structure-Activity Relationship Models. Organ Econ Coop Dev (2004), 1–2.

[334] Eriksson L Jaworska J Worth AP Cronin MTD McDowell RM Gramatica P. Methods for Reliability and Uncertainty Assessment and for Applicability Evaluations of Classification- and Regression-Based QSARs. Environ Health Perspect (2003) 111:1361–75. 10.1289/ehp.5758 PMC1241620 12896860

[335] Mathea M Klingspohn W Baumann K. Chemoinformatic Classification Methods and Their Applicability Domain. Mol Inform (2016) 35:160–80. 10.1002/minf.201501019 27492083

[336] Gadaleta D Mangiatordi GF Catto M Carotti A Nicolotti O. Applicability Domain for QSAR Models. Int J Quant Struct Relat (2016) 1:45–63. 10.4018/IJQSPR.2016010102

[337] Netzeva TI Worth A Aldenberg T Benigni R Cronin MTD Gramatica P. Current Status of Methods for Defining the Applicability Domain of (Quantitative) Structure-Activity Relationships. The Report and Recommendations of ECVAM Workshop 52. Altern Lab Anim (2005) 33:155–73. 10.1177/026119290503300209 16180989

[338] Zorn KM Sun S McConnon CL Ma K Chen EK Foil DH. A Machine Learning Strategy for Drug Discovery Identifies Anti-Schistosomal Small Molecules. ACS Infect Dis (2021) 7:406–20. 10.1021/acsinfecdis.0c00754 PMC7887754 33434015

[339] Ciallella HL Zhu H. Advancing Computational Toxicology in the Big Data Era by Artificial Intelligence: Data-Driven and Mechanism-Driven Modeling for Chemical Toxicity. Chem Res Toxicol (2019) 32:536–47. 10.1021/acs.chemrestox.8b00393 PMC6688471 30907586

[340] LeCun Y Bengio Y Hinton G. Deep Learning. Nature (2015) 521:436–44. 10.1038/nature14539 26017442

[341] Klambauer G Hochreiter S Rarey M. Machine Learning in Drug Discovery. J Chem Inf Model (2019) 59:945–6. 10.1021/acs.jcim.9b00136 30905159

[342] Yu CH Qin Z Martin-Martinez FJ Buehler MJ. A Self-Consistent Sonification Method to Translate Amino Acid Sequences Into Musical Compositions and Application in Protein Design Using Artificial Intelligence. ACS Nano (2019) 13:7471–82. 10.1021/acsnano.9b02180 31240912

[343] Bjerrum EJ. SMILES Enumeration as Data Augmentation for Neural Network Modeling of Molecules (2017). Available at: http://arxiv.org/abs/1703.07076.

[344] Klebe G. Virtual Ligand Screening: Strategies, Perspectives and Limitations. Drug Discov Today (2006) 11:580–94. 10.1016/j.drudis.2006.05.012 PMC7108249 16793526

[345] Braga RC Alves VM Silva AC Liao LM Andrade CH. Virtual Screening Strategies in Medicinal Chemistry: The State of the Art and Current Challenges. Curr Top Med Chem (2014) 14:1899–912. 10.2174/1568026614666140929120749 25262801

[346] Badrinarayan P Sastry GN. Virtual High Throughput Screening in New Lead Identification. Comb Chem High Throughput Screen (2011) 14:840–60. 10.2174/138620711797537102 21843146

[347] Maia EHB Assis LC de Oliveira TA da Silva AM Taranto AG. Structure-Based Virtual Screening: From Classical to Artificial Intelligence. Front Chem (2020) 8:343. 10.3389/fchem.2020.00343 32411671 PMC7200080

[348] Schmidhuber J. Deep Learning in Neural Networks: An Overview. Neural Netw (2015) 61:85–117. 10.1016/j.neunet.2014.09.003 25462637

[349] Valueva MV Nagornov NN Lyakhov PA Valuev GV Chervyakov NI. Application of the Residue Number System to Reduce Hardware Costs of the Convolutional Neural Network Implementation. Math Comput Simul (2020) 177:232–43. 10.1016/j.matcom.2020.04.031

[350] Hadsell R Chopra S LeCun Y. Dimensionality Reduction by Learning an Invariant Mapping. Proc IEEE Comput Soc Conf Comput Vis Pattern Recognit (2006) 2:1735–42. 10.1109/CVPR.2006.100

[351] Hinton GE Salakhutdinov RR. Reducing the Dimensionality of. Science (2006) 313:504–7. 10.1126/science.1127647 16873662

[352] Bender A Cortes-Ciriano I. Artificial Intelligence in Drug Discovery: What is Realistic, What are Illusions? Part 2: A Discussion of Chemical and Biological Data. Drug Discov Today (2021). 10.1016/j.drudis.2020.11.037. PMC8132984 33508423

[353] Lane TR Foil DH Minerali E Urbina F Zorn KM Ekins S. Bioactivity Comparison Across Multiple Machine Learning Algorithms Using Over 5000 Datasets for Drug Discovery. Mol Pharm (2021) 18:403–15. 10.1021/acs.molpharmaceut.0c01013 PMC8237624 33325717

[354] Bajorath J Kearnes S Walters WP Meanwell NA Georg GI Wang S. Artificial Intelligence in Drug Discovery: Into the Great Wide Open. J Med Chem (2020) 63:8651–2. 10.1021/acs.jmedchem.0c01077 32639156

[355] Schneider G Fechner U. Computer-Based De Novo Design of Drug-Like Molecules. Nat Rev Drug Discov (2005) 4:649–63. 10.1038/nrd1799 16056391

[356] Schneider P Schneider G. De Novo Design at the Edge of Chaos. J Med Chem (2016) 59:4077–86. 10.1021/acs.jmedchem.5b01849 26881908

[357] Schneider G Clark DE. Automated De Novo Drug Design: Are We Nearly There Yet? Angew Chem Int Ed (2019) 58:10792–803. 10.1002/anie.201814681 30730601

[358] Chen H Engkvist O. Has Drug Design Augmented by Artificial Intelligence Become a Reality? Trends Pharmacol Sci (2019) 40:806–9. 10.1016/j.tips.2019.09.004 31629547

[359] Sanchez-Lengeling B Aspuru-Guzik A. Inverse Molecular Design Using Machine Learning:Generative Models for Matter Engineering. Science (2018) 361:360–5. 10.1126/science.aat2663 30049875

[360] Segler MHS Kogej T Tyrchan C Waller MP. Generating Focused Molecule Libraries for Drug Discovery With Recurrent Neural Networks. ACS Cent Sci (2018) 4:120–31. 10.1021/acscentsci.7b00512 PMC5785775 29392184

[361] Olivecrona M Blaschke T Engkvist O Chen H. Molecular De-Novo Design Through Deep Reinforcement Learning. J Cheminform (2017) 9:1–14. 10.1186/s13321-017-0235-x 29086083 PMC5583141

[362] Putin E Asadulaev A Ivanenkov Y Aladinskiy V Sanchez-Lengeling B Aspuru-Guzik A. Reinforced Adversarial Neural Computer for De Novo Molecular Design. J Chem Inf Model (2018) 58:1194–204. 10.1021/acs.jcim.7b00690 29762023

[363] Gómez-Bombarelli R Wei JN Duvenaud D Hernández-Lobato JM Sánchez-Lengeling B Sheberla D. Automatic Chemical Design Using a Data-Driven Continuous Representation of Molecules. ACS Cent Sci (2018) 4:268–76. 10.1021/acscentsci.7b00572 PMC5833007 29532027

[364] Sattarov B Baskin II Horvath D Marcou G Bjerrum EJ Varnek A. De Novo Molecular Design by Combining Deep Autoencoder Recurrent Neural Networks With Generative Topographic Mapping. J Chem Inf Model (2019) 59:1182–96. 10.1021/acs.jcim.8b00751 30785751

[365] Yasonik J. Multiobjective De Novo Drug Design With Recurrent Neural Networks and Nondominated Sorting. J Cheminform (2020) 12:14. 10.1186/s13321-020-00419-6 33430996 PMC7026957

[366] Walters WP Murcko M. Assessing the Impact of Generative AI on Medicinal Chemistry. Nat Biotechnol (2020) 38:143–5. 10.1038/s41587-020-0418-2 32001834

[367] Xu Y Lin K Wang S Wang L Cai C Song C. Deep Learning for Molecular Generation. Future Med Chem (2019) 11:567–97. 10.4155/fmc-2018-0358 30698019

[368] Arús-Pous J Patronov A Bjerrum EJ Tyrchan C Reymond JL Chen H. SMILES-Based Deep Generative Scaffold Decorator for De-Novo Drug Design. J Cheminform (2020) 12:1–18. 10.1186/s13321-020-00441-8 33431013 PMC7260788

[369] Ståhl N Falkman G Karlsson A Mathiason G Boström J. Deep Reinforcement Learning for Multiparameter Optimization in De Novo Drug Design. J Chem Inf Model (2019) 59:3166–76. 10.1021/acs.jcim.9b00325 31273995

[370] Zhavoronkov A Ivanenkov YA Aliper A Veselov MS Aladinskiy VA Aladinskaya AV. Deep Learning Enables Rapid Identification of Potent DDR1 Kinase Inhibitors. Nat Biotechnol (2019) 37:1038–40. 10.1038/s41587-019-0224-x 31477924

[371] Zhavoronkov A Aspuru-Guzik A. Reply to ‘Assessing the Impact of Generative AI on Medicinal Chemistry’. Nat Biotechnol (2020) 38:146–6. 10.1038/s41587-020-0417-3 32001835

[372] Bush JT Pogany P Pickett SD Barker M Baxter A Campos S. A Turing Test for Molecular Generators. J Med Chem (2020) 63:11964–71. 10.1021/acs.jmedchem.0c01148 32955254

[373] Brown N Fiscato M Segler MHS Vaucher AC. GuacaMol: Benchmarking Models for De Novo Molecular Design. J Chem Inf Model (2019) 59:1096–108. 10.1021/acs.jcim.8b00839 30887799

[374] Polykovskiy D Zhebrak A Sanchez-Lengeling B Golovanov S Tatanov O Belyaev S. Molecular Sets (MOSES): A Benchmarking Platform for Molecular Generation Models. Front Pharmacol (2020) 11:565644. 10.3389/fphar.2020.565644 33390943 PMC7775580

[375] Gao W Coley CW. The Synthesizability of Molecules Proposed by Generative Models. J Chem Inf Model (2020) 60:5714–23. 10.1021/acs.jcim.0c00174 32250616

[376] Corey E Long A Rubenstein S. Computer-Assisted Analysis in Organic Synthesis. Science (1985) 228:408–18. 10.1126/science.3838594 3838594

[377] Zheng S Rao J Zhang Z Xu J Yang Y. Predicting Retrosynthetic Reactions Using Self-Corrected Transformer Neural Networks. J Chem Inf Model (2020) 60:47–55. 10.1021/acs.jcim.9b00949 31825611

[378] Gelernter HL Sanders AF Larsen DL Agarwal KK Boivie RH Spritzer GA. Empirical Explorations of SYNCHEM. Science (80- ) (1977) 197:1041–9. 10.1126/science.197.4308.1041 17836062

[379] Huang Q Li L-L Yang S-Y. RASA: A Rapid Retrosynthesis-Based Scoring Method for the Assessment of Synthetic Accessibility of Drug-like Molecules. J Chem Inf Model (2011) 51:2768–77. 10.1021/ci100216g 21932860

[380] Jorgensen WL Laird ER Gushurst AJ Fleischer JM Gothe SA Helson HE. CAMEO: A Program for the Logical Prediction of the Products of Organic Reactions. Pure Appl Chem (1990) 62:1921–32. 10.1351/pac199062101921

[381] Satoh H Funatsu K. SOPHIA, a Knowledge Base-Guided Reaction Prediction System - Utilization of a Knowledge Base Derived From a Reaction Database. J Chem Inf Model (1995) 35:34–44. 10.1021/ci00023a005

[382] Röse P Gasteiger J. EROS 6.0, a Knowledge Based System for Reaction Prediction — Application to the Regioselectivity of the Diels-Alder Reaction. In: Software Development in Chemistry 4. Berlin, Heidelberg: Springer Berlin Heidelberg (1990). p. 275–88. 10.1007/978-3-642-75430-2_30

[383] Szymkuć S Gajewska EP Klucznik T Molga K Dittwald P Startek M. Computer-Assisted Synthetic Planning: The End of the Beginning. Angew Chem Int Ed (2016) 55:5904–37. 10.1002/anie.201506101 27062365

[384] Nair VH Schwaller P Laino T. Data-Driven Chemical Reaction Prediction and Retrosynthesis. Chimia (Aarau) (2019) 73:997–1000. 10.2533/chimia.2019.997 31883550

[385] Struble TJ Alvarez JC Brown SP Chytil M Cisar J Desjarlais RL. Current and Future Roles of Artificial Intelligence in Medicinal Chemistry Synthesis. J Med Chem (2020) 63:8667–82. 10.1021/acs.jmedchem.9b02120 PMC7457232 32243158

[386] Coley CW Green WH Jensen KF. Machine Learning in Computer-Aided Synthesis Planning. Acc Chem Res (2018) 51:1281–9. 10.1021/acs.accounts.8b00087 29715002

[387] IBM RXN Platform . Available at: https://rxn.res.ibm.com/ (Accessed May 5, 2021).

[388] Badowski T Gajewska EP Molga K Grzybowski BA. Synergy Between Expert and Machine-Learning Approaches Allows for Improved Retrosynthetic Planning. Angew Chem Int Ed (2020) 59:725–30. 10.1002/anie.201912083 31750610

